# Supplementary material for: Pharmacological Effects of Agastache rugosa against Gastritis Using a Network Pharmacology Approach
Source: Biomolecules. 2020 Sep 9;10(9):1298. doi: 10.3390/biom10091298 (PMC7565599; doi:10.3390/biom10091298)
Supplement: Supplementary file 1 [file biomolecules-10-01298-s001.zip › gastritis_supplementary_table1.pdf]

Supplementary table 1. Chemical components in Agastache rugosa through ADME screening

No.	Sources	Chemical names	Mol ID	MW	AlogP	Hdon	Hacc	OB (%)	Caco-2	BBB	DL	FASA-	TPSA	RBN	HL
1	Isolation	Luteolin	MOL000006	286.25	2.07	4	6	36.16	0.19	-0.84	0.25	0.39	111.13	1	15.94
2	Isolation	Apigetrin	MOL000007	432.41	0.43	6	10	9.68	-1.08	-2.26	0.74	0.32	170.05	4	
	korenaTK	Apigetrin	MOL000007	432.41	0.43	6	10	9.68	-1.08	-2.26	0.74	0.32	170.05	4	
3	Isolation	Apigenin	MOL000008	270.25	2.33	3	5	23.06	0.43	-0.61	0.21	0.41	90.9	1	
	TM-MC	Apigenin	MOL000008	270.25	2.33	3	5	23.06	0.43	-0.61	0.21	0.41	90.9	1	
4	Isolation	Luteoloside	MOL000009	448.41	0.16	7	11	7.29	-1.23	-2.49	0.78	0.32	190.28	4	
5	TM-MC	Limonene	MOL000023	136.26	3.5	0	0	39.84	1.83	2.12	0.02	0	0	1	11.68
6	TM-MC	alpha-caryophyllene	MOL000024	204.39	5.04	0	0	22.98	1.88	2.08	0.06	0	0	0	
7	TM-MC	Caryophyllene	MOL000036	204.39	4.75	0	0	29.7	1.83	2.07	0.09	0	0	0	
8	TM-MC	gamma-elemene (=elixene)	MOL000037	204.39	4.93	0	0	23.79	1.87	2.1	0.06	0	0	2	
9	TM-MC	Palmitic acid (=hexadecanoic acid)	MOL000069	256.48	6.37	1	2	19.3	1.09	1	0.1	0	37.3	14	
10	korenaTK	para-Cymene	MOL000117	134.24	3.51	0	0	27.2	1.86	2.1	0.02	0.34	0	1	
11	TM-MC	alpha-pinene	MOL000125	136.26	2.87	0	0	46.25	1.85	2.3	0.05	0.25	0	0	11.42
	korenaTK	alpha-Pinene(+,-)	MOL000125	136.26	2.87	0	0	46.25	1.85	2.3	0.05	0.25	0	0	11.42
12	TM-MC	beta-pinene	MOL000126	136.26	2.93	0	0	44.84	1.8	2.12	0.05	0.27	0	0	11.32
	korenaTK	beta-Pinene-(+)	MOL000126	136.26	2.93	0	0	44.84	1.8	2.12	0.05	0.27	0	0	11.32
13	TM-MC	Linoleic acid	MOL000131	280.5	6.39	1	2	41.9	1.16	0.9	0.14	0.25	37.3	14	7.5
14	TM-MC	gamma-terpinene	MOL000202	136.26	3.45	0	0	33.02	1.88	2.05	0.02	0.27	0	1	11.08
15	korenaTK	Methyleugenol	MOL000207	178.25	2.81	0	2	73.36	1.47	1.41	0.04	0.27	18.46	4	2.92
	TM-MC	Methyleugenol	MOL000207	178.25	2.81	0	2	73.36	1.47	1.41	0.04	0.27	18.46	4	2.92
16	TM-MC	Aromadendrene	MOL000208	204.39	4.22	0	0	55.74	1.81	2.06	0.1	0.26	0	0	11.84
17	TM-MC	alpha-terpineol	MOL000232	154.28	2.42	1	1	46.3	1.28	1.4	0.03	0.26	20.23	1	10.75
18	TM-MC	Eugenol	MOL000254	164.22	2.55	1	2	56.24	1.35	1.32	0.04	0.32	29.46	3	0.92
19	TM-MC	Carvacrol	MOL000259	150.24	3.24	1	1	43.28	1.58	1.71	0.03	0.33	20.23	1	11.29
20	korenaTK	Oleanolic acid	MOL000263	456.78	6.42	2	3	29.02	0.59	0.07	0.76	0.25	57.53	1	
21	TM-MC	Caffeic acid	MOL000414	180.17	1.37	3	4	54.97	0.27	0.11	0.05	0	77.76	2	1.63
22	TM-MC	Calycosin	MOL000417	284.28	2.32	2	5	47.75	0.52	-0.43	0.24	0	79.9	2	17.1
23	korenaTK	Anethole	MOL000475	148.22	2.77	0	1	32.49	1.75	1.81	0.03	0	9.23	2	1.68
24	korenaTK	beta-Farnesene	MOL000479	204.39	5.52	0	0	17.42	1.95	2.21	0.05	0	0	7	
25	TM-MC	Genistein	MOL000481	270.25	2.07	3	5	17.93	0.43	-0.4	0.21	0	90.9	1	
26	TM-MC	Kaempferol 3-O-glucoside	MOL000561	448.41	-0.32	7	11	14.03	-1.34	-1.97	0.74	0.34	190.28	4	
27	TM-MC	beta-bourbonene	MOL000611	204.39	4.22	0	0	16.98	1.82	2.11	0.11	0.27	0	1	
28	TM-MC	para-menthan-3-one	MOL000715	154.28	2.6	0	1	57.9	1.35	1.7	0.03	0.24	17.07	1	10.57
29	korenaTK	1,3,11-Elematriene (=beta-elemene)	MOL000908	204.39	4.79	0	0	25.63	1.84	2.07	0.06	0.33	0	3	
	TM-MC	beta-elemene	MOL000908	204.39	4.79	0	0	25.63	1.84	2.07	0.06	0.33	0	3	
30	korenaTK	Linalool	MOL000920	154.28	2.74	1	1	38.29	1.29	1.33	0.02	0.32	20.23	4	6.29
	TM-MC	Linalool	MOL000920	154.28	2.74	1	1	38.29	1.29	1.33	0.02	0.32	20.23	4	6.29
31	TM-MC	cis-alpha-farnesene	MOL000930	204.39	5.46	0	0	8.15	1.92	1.85	0.05	0.35	0	6	
32	TM-MC	alpha-farnesene	MOL000932	204.39	5.46	0	0	21.7	1.97	1.89	0.05	0.32	0	6	
33	TM-MC	Germacrene B	MOL000936	204.39	5.47	0	0	18.69	1.89	2.07	0.06	0.29	0	0	
34	TM-MC	Isoledene	MOL000937	204.39	4.36	0	0	49.01	1.82	2.08	0.1	0.22	0	0	12.47
35	TM-MC	alpha-muurolene	MOL001123	204.39	4.75	0	0	19.5	1.84	2.16	0.08	0.25	0	1	
36	TM-MC	gamma-muurolene	MOL001180	204.39	4.8	0	0	21.53	1.84	2.05	0.08	0.25	0	1	
37	TM-MC	Bicyclogermacrene	MOL001184	204.39	4.7	0	0	30.73	1.86	2.13	0.08	0.25	0	0	5.62
38	TM-MC	Caryophyllene oxide	MOL001193	235.38	2.17	0	2	45.75	1.09	1.27	0.15	0.03	35.59	0	7.08
39	korenaTK	beta-Sitosterol	MOL001488	414.79	8.08	1	1	6.59	1.42	1.16	0.75	0.2	20.23	6	
40	TM-MC	Acetophenone	MOL001578	120.16	1.57	0	1	48.19	1.36	1.54	0.02	0.45	17.07	1	25.2
41	TM-MC	alpha-cadinol	MOL001599	208.38	3.28	1	1	64.81	1.32	1.43	0.09	0.26	20.23	0	7.24
42	Isolation	Phlorizin	MOL001623	436.45	0.75	7	10	2.88	-1.23	-2.02	0.6	0.34	177.14	7	
43	Isolation	Acacetin	MOL001689	284.28	2.59	2	5	34.97	0.67	-0.05	0.24	0.35	79.9	2	17.25
	korenaTK	Acacetin	MOL001689	284.28	2.59	2	5	34.97	0.67	-0.05	0.24	0.35	79.9	2	17.25
	TM-MC	Acacetin	MOL001689	284.28	2.59	2	5	34.97	0.67	-0.05	0.24	0.35	79.9	2	17.25
44	TM-MC	3-O-Caffeoylquinic acid (=chlorogenic acid)	MOL001955	354.34	-0.42	6	9	11.93	-1.03	-1.71	0.33	0.37	164.75	5	
45	TM-MC	Pulegone	MOL001972	152.26	2.75	0	1	51.6	1.39	1.74	0.03	0.26	17.07	0	11.19
46	korenaTK	Acetyl oleanolic aldehyde	MOL002001	498.82	6.8	1	4	14.24	0.65	0.13	0.7	0.24	63.6	3	
47	TM-MC	Thymol	MOL002042	150.24	3.24	1	1	41.47	1.6	1.68	0.03	0.33	20.23	1	11.33
48	TM-MC	alpha-cubebene	MOL002085	204.39	4.17	0	0	16.73	1.83	2.1	0.11	0.25	0	1	
49	TM-MC	Spatulenol (=1H-Cycloprop(e)azulen-7-ol)	MOL002153	220.39	3.01	1	1	82.33	1.37	1.49	0.12	0.28	20.23	0	12.04
50	korenaTK	Estragole	MOL002361	148.22	2.82	0	1	36.59	1.72	1.83	0.03	0.34	9.23	3	1.79
	TM-MC	Estragole (=methylchavicol)	MOL002361	148.22	2.82	0	1	36.59	1.72	1.83	0.03	0.34	9.23	3	1.79
51	korenaTK	Anisaldehyde	MOL002836	136.16	1.57	0	2	21.54	1.12	1.15	0.02	0.33	26.3	2	
52	Isolation	Diosmetin	MOL002881	300.28	2.32	3	6	31.14	0.46	-0.66	0.27	0.34	100.13	2	16.34
53	Isolation	Ethyl caffeate	MOL002902	208.23	1.97	2	4	103.85	0.73	0.51	0.07	0.34	66.76	4	3.76
54	TM-MC	Germacrene D	MOL003127	204.39	5.14	0	0	19.22	1.83	2.02	0.06	0	0	1	
55	TM-MC	tau-muurolol	MOL003537	222.41	3.78	1	1	30.41	1.36	1.44	0.09	0.23	20.23	1	7.11
56	korenaTK	delta-Cadinene	MOL003556	204.39	4.94	0	0	20.29	1.83	2.1	0.08	0	0	1	
	TM-MC	delta-Cadinene	MOL003556	204.39	4.94	0	0	20.29	1.83	2.1	0.08	0	0	1	
57	TM-MC	Esculetin	MOL003837	178.15	1.37	2	4	22.97	0.44	0.02	0.07	0.36	70.67	0	
58	TM-MC	Cuminic alcohol	MOL003948	150.24	2.42	1	1	42.57	1.2	1.24	0.03	0.3	20.23	2	1.58
59	korenaTK	Calamenene-(-)	MOL004079	202.37	5.04	0	0	17.31	1.89	2.1	0.08	0	0	1	
60	TM-MC	Viridiflorol	MOL004419	222.41	3.2	1	1	19.94	1.31	1.43	0.12	0	20.23	0	
61	Isolation	Anisic acid	MOL005125	152.16	1.42	1	3	29.69	0.69	0.51	0.03	0.36	46.53	2	
62	korenaTK	Maslinic acid	MOL005559	472.78	5.46	3	4	15.54	0.1	-0.55	0.74	0.25	77.76	1	
63	TM-MC	Morrilol (=1-octen-3-ol)	MOL005729	416.62	5.91	4	7	15.22	-0.32	-0.73	0.4	0	132.13	20	
64	Isolation	Nepetoidin B (= [(Z)-2-(3,4-dihydroxyphenyl)vinyl] (E)-3-(3,4-dihydroxyphenyl)pr	MOL006191	314.31	2.6	4	6	1.32	0.31	-0.73	0.28	0.43	107.22	5	
65	Isolation	Methyl caffeate	MOL006218	194.2	1.62	2	4	30.68	0.54	0.06	0.06	0.36	66.76	3	2.9
66	Isolation	Methyl rosmarinate	MOL007116	374.37	2.94	4	8	1.37	0	-0.72	0.37	0.35	133.52	8	
67	korenaTK	gamma-Cadinene-(+)	MOL008940	204.39	4.8	0	0	21.34	1.85	2.09	0.08	0.27	0	1	
68	Isolation	Rosmarinic acid	MOL011865	360.34	2.69	5	8	1.38	-0.54	-1.24	0.35	0.47	144.52	7	
	korenaTK	Rosmarinic acid	MOL011865	360.34	2.69	5	8	1.38	-0.54	-1.24	0.35	0.47	144.52	7	
	TM-MC	Rosmarinic acid	MOL011865	360.34	2.69	5	8	1.38	-0.54	-1.24	0.35	0.47	144.52	7	
69	Isolation	Tilianin	MOL011878	446.44	0.68	5	10	19.66	-0.82	-2.09	0.79	0.28	159.05	5	
	korenaTK	Tilianin	MOL011878	446.44	0.68	5	10	19.66	-0.82	-2.09	0.79	0.28	159.05	5	
	TM-MC	Tilianin	MOL011878	446.44	0.68	5	10	19.66	-0.82	-2.09	0.79	0.28	159.05	5	
70	Isolation	Ethyl Rosmarinate	MOL012205	388.4	3.29	4	8	1.39	0.12	-0.74	0.4	0.36	133.52	9	
71	korenaTK	Daucosterol	MOL012237	576.95	6.34	4	6	20.63	0.03	-0.61	0.63	0.19	99.38	9	
72	korenaTK	para-Methoxycinnamaldehyde	MOL013205	162.2	1.93	0	2	59.64	1.12	0.98	0.04	0.39	26.3	3	4.34
73	Isolation	Citrusin C	MOL013429	326.38	0.65	4	7	17.59	-0.49	-0.99	0.23	0.29	108.61	6	
74	Isolation	(2S)-Poncirenin													
75	Isolation	(6R,7E,9R)-9-Hydroxy-megastigma-4,7-dien-3-one 9-O-β-D-glucopyranoside													
76	korenaTK	19(4-3)Abeo-12,14,15-trihydroxy-11-methoxyabiet-4(18),8,11,13-Tetraen-7-one													
77	Isolation	1-Hydroxy-2-O-β-D-glucopyranosyl-4-allylbenzene													
78	Isolation	1-O-β-D-Glucopyranosyl-2-hydroxy-4-allylbenzene													
79	TM-MC	2,5-Dihydroxycinnamic acid													
80	korenaTK	5-Allyl-2,3-dimethoxyphenol													
81	TM-MC	6,7-Dimethoxyquercetin 3-O-glucopyranoside													
82	Isolation	Acacetin 7-O-(3-O-acetyl-β-D-glucopyranoside)													
83	Isolation	Acacetin-7-O-(2"-O-acetyl-6"-O-malonyl)-β-D-glucopyranoside													
84	Isolation	Acacetin-7-O-glucoside-6"-O-malonyl ester													
85	korenaTK	Agastachin													
86	korenaTK	Agastachoside													
87	korenaTK	Agastaquinone													
88	TM-MC	Cadina-4,9-diene													
89	TM-MC	Calycosin isomer													
90	Isolation	Chavicol beta-D-glucopyranoside													
91	korenaTK	Dehydroagastanol													
92	Isolation	Diosmetin-7-O-beta-Dglucopyranoside													
	korenaTK	Diosmetin-7-O-beta-D-glucopyranoside													
93	TM-MC	Emodin 3-O-glucoside													
94	korenaTK	Erythrodiol-3-acetate													
95	TM-MC	Fraxiresinol hexoside													
96	Isolation	Isoagastachoside													
	korenaTK	Isoagastachoside													
97	TM-MC	Octahydro-7-methyl-methylene-4-(1-methylethyl)-1H-cyclopenta [1, 3] cyclopropa [1, 2] benzene													
98	TM-MC	Phloretin-hexoside													
99	TM-MC	Rosmarinic acid isomer													
