# Supplementary material for: Pharmacological Effects of Agastache rugosa against Gastritis Using a Network Pharmacology Approach
Source: Biomolecules. 2020 Sep 9;10(9):1298. doi: 10.3390/biom10091298 (PMC7565599; doi:10.3390/biom10091298)
Supplement: Supplementary file 1 [file biomolecules-10-01298-s001.zip › gastritis_supplementary_table2.pdf]

Supplementary table 2. All genes related chemical components based on STITCH DB

Chemical	gene	combined score
Diosmetin	ABCC1	0.763
Luteolin	ABCC1	0.714
Luteolin	ADAMTS3	0.8
Luteolin	ADAMTS4	0.8
Luteolin	AGT	0.786
Luteolin	AKT1	0.856
Luteolin	AURKB	0.8
Luteolin	BCL2L1	0.841
Calycosin	BLVRB	0.845
Luteolin	C3	0.786
Luteolin	CASP3	0.947
Luteolin	CASP7	0.7
Luteolin	CASP9	0.7
Luteolin	CCNA2	0.856
Rosmarinic acid	CCR3	0.8
Luteolin	CDH1	0.8
Luteolin	CDK2	0.942
Luteolin	CDK4	0.8
Luteolin	CSNK2A1	0.701
Luteolin	CSNK2A2	0.701
Luteolin	CYP19A1	0.8
Diosmetin	CYP1A1	0.88
Acacetin	CYP1A1	0.869
Luteolin	CYP1A1	0.777
Acacetin	CYP1A2	0.869
Acacetin	CYP1B1	0.905
Diosmetin	CYP1B1	0.887
Luteolin	CYP1B1	0.847
Diosmetin	CYP2C8	0.8
Luteolin	E2F5	0.8
Luteolin	EGFR	0.869
Luteolin	ERBB2	0.786
Luteolin	ESR2	0.7
Luteolin	EZH2	0.818
Luteolin	FN1	0.8
Luteolin	FOS	0.944
Rosmarinic acid	FOS	0.8
Luteolin	FOSB	0.7
Luteolin	FOXO1	0.817
Calycosin	HMGB1	0.824

Luteolin	HMOX1	0.822
Luteolin	HSP90AA1	0.8
Luteolin	IGF1	0.8
Luteolin	IGF2	0.8
Rosmarinic acid	IKBKB	0.8
Acacetin	IL13	0.8
Luteolin	IL1B	0.8
Rosmarinic acid	IL2	0.8
Acacetin	IL5	0.8
Luteolin	IRS1	0.8
Luteolin	JUN	0.946
Acacetin	JUN	0.8
Luteolin	JUNB	0.7
Luteolin	JUND	0.7
Rosmarinic acid	LCK	0.8
Luteolin	LCN2	0.8
Luteolin	MAP3K8	0.8
Calycosin	MAPK1	0.7
Luteolin	MAPK1	0.7
Luteolin	MAPK10	0.7
Calycosin	MAPK3	0.7
Luteolin	MAPK3	0.7
Luteolin	MAPK8	0.951
Luteolin	MAPK9	0.731
Luteolin	MMP9	0.949
Luteolin	MTOR	0.823
Luteolin	NFE2L2	0.816
Luteolin	NOS1	0.786
Luteolin	NOS2	0.784
Acacetin	NR1I2	0.7
Rosmarinic acid	PARG	0.7
Luteolin	PCK1	0.786
Luteolin	PKM	0.726
Diosmetin	PKM	0.7
Luteolin	PPARG	0.818
Rosmarinic acid	PROCR	0.7
Luteolin	PTK2	0.7
Luteolin	RPS6KA1	0.8
Luteolin	RPS6KA2	0.786
Luteolin	RPS6KA3	0.8
Acacetin	SELE	0.8
Luteolin	SMAD2	0.859
Acacetin	STAT1	0.82

Luteolin	STAT3	0.748
Luteolin	TAF9	0.8
Luteolin	TBK1	0.7
Luteolin	TLR4	0.817
Luteolin	TLR5	0.8
Luteolin	TNFRSF10B	0.8
Luteolin	TNKS2	0.8
Luteolin	TOP1	0.846
Luteolin	TP53	0.828
Luteolin	TTR	0.8
Calycosin	UGT1A1	0.7
Calycosin	UGT1A10	0.7
Luteolin	UGT1A3	0.729
Calycosin	UGT1A3	0.7
Calycosin	UGT1A7	0.7
Calycosin	UGT1A8	0.7
Calycosin	UGT1A9	0.7
Calycosin	UGT2B7	0.7
Luteolin	USP8	0.8
Luteolin	VEGFA	0.855
Acacetin	VEGFA	0.8
Luteolin	VRK1	0.8
Luteolin	TNF	0.698
Luteolin	PCK2	0.665
Luteolin	BCAM	0.664
Luteolin	PDZD4	0.642
Luteolin	NOS3	0.613
Luteolin	ERBB3	0.579
Luteolin	ERBB4	0.579
Luteolin	CNTN6	0.574
Luteolin	C4A	0.566
Luteolin	C4B	0.566
Luteolin	AKR1B1	0.535
Luteolin	C5	0.515
Luteolin	MTRR	0.473
Luteolin	POR	0.473
Luteolin	XDH	0.463
Luteolin	LAMA5	0.44
Luteolin	MCAM	0.431
Luteolin	A2M	0.423
Luteolin	A2ML1	0.423
Luteolin	COQ6	0.386
Luteolin	KMO	0.386

Luteolin	SQLE	0.386
Luteolin	FUT2	0.374
Rosmarinic acid	AKR1B1	0.369
Luteolin	LTA	0.366
Luteolin	DCP2	0.363
Luteolin	NOX4	0.363
Luteolin	NUDT1	0.363
Luteolin	NUDT10	0.363
Luteolin	NUDT11	0.363
Luteolin	NUDT14	0.363
Luteolin	NUDT2	0.363
Luteolin	NUDT5	0.363
Luteolin	NUDT7	0.363
Luteolin	NUDT8	0.363
Luteolin	NDOR1	0.344
Luteolin	TYW1	0.344
Acacetin	RGS17	0.336
Luteolin	MAP2K4	0.332
Luteolin	UCN2	0.332
Luteolin	CCNE2	0.331
Luteolin	HMSD	0.32
Luteolin	PTS	0.32
Luteolin	SERPINA1	0.32
Luteolin	SERPINA10	0.32
Luteolin	SERPINA11	0.32
Luteolin	SERPINA12	0.32
Luteolin	SERPINA3	0.32
Luteolin	SERPINA4	0.32
Luteolin	SERPINA5	0.32
Luteolin	SERPINA6	0.32
Luteolin	SERPINA7	0.32
Luteolin	SERPINA9	0.32
Luteolin	SERPINB1	0.32
Luteolin	SERPINB10	0.32
Luteolin	SERPINB12	0.32
Luteolin	SERPINB13	0.32
Luteolin	SERPINB2	0.32
Luteolin	SERPINB3	0.32
Luteolin	SERPINB4	0.32
Luteolin	SERPINB5	0.32
Luteolin	SERPINB6	0.32
Luteolin	SERPINB7	0.32
Luteolin	SERPINB8	0.32

Luteolin	SERPINB9	0.32
Luteolin	SERPINC1	0.32
Luteolin	SERPIND1	0.32
Luteolin	SERPINE1	0.32
Luteolin	SERPINE2	0.32
Luteolin	SERPINE3	0.32
Luteolin	SERPINF1	0.32
Luteolin	SERPINF2	0.32
Luteolin	SERPING1	0.32
Luteolin	SERPINH1	0.32
Luteolin	SERPINI1	0.32
Luteolin	SERPINI2	0.32
Luteolin	SMAD1	0.316
Luteolin	SMAD3	0.316
Luteolin	SMAD5	0.316
Luteolin	SMAD9	0.316
Luteolin	FLT3	0.312
Luteolin	CAT	0.307
Luteolin	CRHR2	0.305
Luteolin	MPI	0.298
Luteolin	CDC25A	0.289
Acacetin	RGS20	0.288
Acacetin	RPLP1	0.288
Luteolin	AASDHPPT	0.282
Luteolin	AKR1B10	0.281
Luteolin	AKR1E2	0.281
Calycosin	PNPO	0.281
Luteolin	PLK1	0.277
Calycosin	ABHD14B	0.272
Diosmetin	DET1	0.267
Luteolin	HDC	0.267
Luteolin	AKR1A1	0.266
Acacetin	KCNK10	0.266
Acacetin	RGS19	0.266
Tilianin	C1orf116	0.263
Luteolin	CDKN1B	0.262
Acacetin	WIZ	0.257
Luteolin	TOP1MT	0.252
Luteolin	EZR	0.248
Luteolin	MSN	0.248
Luteolin	NF2	0.248
Luteolin	RDX	0.248
Diosmetin	ADA	0.246

Diosmetin	ADAL	0.246
Diosmetin	AMPD1	0.246
Diosmetin	AMPD2	0.246
Diosmetin	AMPD3	0.246
Diosmetin	CECR1	0.246
Calycosin	NKIRAS2	0.244
Luteolin	PRODH	0.242
Luteolin	PRODH2	0.242
Luteolin	HSPE1	0.239
Luteolin	IL6	0.239
Luteolin	KEL	0.239
Luteolin	CCNB1	0.237
Luteolin	AKR1C1	0.236
Luteolin	AKR1C2	0.236
Luteolin	AKR1C3	0.236
Luteolin	AKR1C4	0.236
Luteolin	AKR1D1	0.236
Luteolin	ABCG2	0.235
Luteolin	KARS	0.235
Luteolin	MAOA	0.235
Rosmarinic acid	PPEF1	0.235
Rosmarinic acid	PPEF2	0.235
Rosmarinic acid	PPP2CA	0.235
Rosmarinic acid	PPP2CB	0.235
Rosmarinic acid	PPP3CA	0.235
Rosmarinic acid	PPP3CB	0.235
Rosmarinic acid	PPP3CC	0.235
Rosmarinic acid	PPP4C	0.235
Rosmarinic acid	PPP5C	0.235
Rosmarinic acid	PPP5D1	0.235
Luteolin	EPOR	0.232
Luteolin	SLC23A1	0.232
Luteolin	SLC23A2	0.232
Luteolin	SLC23A3	0.232
Luteolin	SLC4A1	0.231
Luteolin	SLC4A10	0.231
Luteolin	SLC4A2	0.231
Luteolin	SLC4A3	0.231
Luteolin	SLC4A4	0.231
Luteolin	SLC4A5	0.231
Luteolin	SLC4A7	0.231
Luteolin	SLC4A8	0.231
Luteolin	AKR1B15	0.228

Acacetin	CCNB1	0.227
Acacetin	CCNB2	0.227
Acacetin	CCNB3	0.227
Acacetin	CCNG1	0.227
Acacetin	CCNG2	0.227
Acacetin	CCNI	0.227
Acacetin	CCNI2	0.227
Acacetin	CCNO	0.227
Acacetin	CNTD2	0.227
Rosmarinic acid	AMFR	0.226
Rosmarinic acid	BCHE	0.226
Acacetin	KCNK2	0.226
Calycosin	RASL11A	0.226
Luteolin	AKR1CL1	0.225
Luteolin	CBR4	0.224
Luteolin	HSD17B8	0.224
Calycosin	TAGLN2	0.222
Calycosin	NBL1	0.219
Calycosin	RASL11B	0.218
Luteolin	PPCDC	0.215
Luteolin	PPCS	0.215
Luteolin	ABCA1	0.214
Luteolin	ABCA10	0.214
Luteolin	ABCA12	0.214
Luteolin	ABCA13	0.214
Luteolin	ABCA2	0.214
Luteolin	ABCA3	0.214
Luteolin	ABCA4	0.214
Luteolin	ABCA5	0.214
Luteolin	ABCA6	0.214
Luteolin	ABCA7	0.214
Luteolin	ABCA8	0.214
Luteolin	ABCA9	0.214
Luteolin	ABCG1	0.214
Luteolin	ABCG4	0.214
Luteolin	ABCG5	0.214
Luteolin	ABCG8	0.214
Diosmetin	CDKN2C	0.214
Luteolin	ECM1	0.214
Luteolin	DAB2IP	0.213
Luteolin	NCOA1	0.213
Tilianin	PGS1	0.213
Luteolin	CDKN2C	0.212

Rosmarinic acid	FYN	0.212
Luteolin	C1orf106	0.211
Luteolin	CCDC120	0.211
Luteolin	FRMD4A	0.211
Luteolin	FRMD4B	0.211
Luteolin	H3F3B	0.211
Luteolin	PARP1	0.21
Luteolin	IL8	0.208
Calycosin	DIRAS1	0.206
Rosmarinic acid	HTRA1	0.206
Rosmarinic acid	HTRA3	0.206
Rosmarinic acid	HTRA4	0.206
Luteolin	GCLM	0.205
Luteolin	HSPD1	0.203
Diosmetin	HSF5	0.202
Luteolin	MST1R	0.202
Acacetin	RGS4	0.202
Luteolin	SLC4A11	0.2
Luteolin	SLC4A9	0.2
Luteolin	VIM	0.2
Acacetin	DDI1	0.197
Acacetin	DDI2	0.197
Calycosin	NKIRAS1	0.197
Acacetin	NRIP2	0.197
Acacetin	NRIP3	0.197
Luteolin	PUS7	0.197
Luteolin	PUS7L	0.197
Luteolin	SLC6A3	0.197
Acacetin	RGS2	0.195
Acacetin	RPLP2	0.194
Rosmarinic acid	TMPRSS11D	0.194
Luteolin	GPX1	0.193
Luteolin	GSK3B	0.193
Calycosin	DIRAS2	0.192
Luteolin	GPX2	0.192
Acacetin	KCNJ9	0.192
Acacetin	MYB	0.192
Acacetin	MYBL1	0.192
Acacetin	MYBL2	0.192
Acacetin	SNAPC4	0.192
Acacetin	ZER1	0.192
Acacetin	ADORA1	0.191
Calycosin	ALOX15	0.19

Rosmarinic acid	MMP9	0.19
Calycosin	RASD1	0.19
Calycosin	RASL12	0.19
Luteolin	DCAF7	0.189
Diosmetin	EEF2	0.189
Luteolin	BCHE	0.188
Calycosin	PIK3CG	0.188
Luteolin	ALOX5	0.187
Rosmarinic acid	HTRA2	0.184
Rosmarinic acid	PON1	0.183
Rosmarinic acid	PON2	0.183
Rosmarinic acid	PON3	0.183
Luteolin	CETN2	0.182
Luteolin	CETN3	0.182
Rosmarinic acid	ENSA	0.182
Luteolin	SLC6A2	0.182
Luteolin	TIMM50	0.182
Luteolin	GAB1	0.181
Diosmetin	SIK2	0.181
Acacetin	MBTPS2	0.18
Luteolin	PCNA	0.179
Diosmetin	AVP	0.178
Acacetin	CDNF	0.178
Luteolin	HES1	0.178
Luteolin	HES4	0.178
Rosmarinic acid	PID1	0.178
Diosmetin	GUCY1A3	0.177
Acacetin	RRAS	0.177
Acacetin	RRAS2	0.177
Luteolin	CLPP	0.176
Rosmarinic acid	SPICE1	0.176
Rosmarinic acid	TNFRSF14	0.175
Rosmarinic acid	ATP5A1	0.174
Diosmetin	DCAF7	0.174
Acacetin	ISCU	0.174
Luteolin	PIR	0.173
Luteolin	TYR	0.173
Acacetin	CCNA1	0.171
Acacetin	CCNA2	0.171
Acacetin	CCNF	0.171
Luteolin	ETFB	0.171
Rosmarinic acid	GLA	0.171
Rosmarinic acid	NAGA	0.171

Calycosin	PLA2G4A	0.17
Rosmarinic acid	SCO1	0.17
Rosmarinic acid	SCO2	0.17
Luteolin	PTGS2	0.169
Rosmarinic acid	SPEN	0.169
Acacetin	ANKRD45	0.168
Luteolin	PGR	0.168
Acacetin	SLC9C2	0.168
Tilianin	ENO1	0.167
Tilianin	ENO2	0.167
Tilianin	ENO3	0.167
Luteolin	SI	0.167
Luteolin	STIL	0.167
Diosmetin	AHRR	0.166
Luteolin	GSR	0.166
Acacetin	KCNJ5	0.166
Tilianin	LCT	0.166
Luteolin	PSMD1	0.166
Luteolin	TIMM50	0.166
Luteolin	NRIP1	0.163
Luteolin	NUBPL	0.163
Acacetin	TP53INP2	0.163
Diosmetin	TRAT1	0.163
Tilianin	ENO4	0.162
Luteolin	ALAD	0.161
Luteolin	EPS8	0.161
Luteolin	EPS8L2	0.161
Diosmetin	NTHL1	0.161
Luteolin	SLC6A4	0.161
Rosmarinic acid	XRCC3	0.161
Luteolin	HIST1H4A	0.16
Luteolin	HIST1H4B	0.16
Luteolin	HIST1H4C	0.16
Luteolin	HIST1H4D	0.16
Luteolin	HIST1H4E	0.16
Luteolin	HIST1H4F	0.16
Luteolin	HIST1H4H	0.16
Luteolin	HIST1H4I	0.16
Luteolin	HIST1H4J	0.16
Luteolin	HIST1H4K	0.16
Luteolin	HIST1H4L	0.16
Luteolin	HIST2H4A	0.16
Luteolin	HIST2H4B	0.16

Luteolin	HIST4H4	0.16
Rosmarinic acid	JUN	0.16
Rosmarinic acid	MCL1	0.159
Luteolin	NR1H2	0.159
Luteolin	NR1H3	0.159
Rosmarinic acid	TYSND1	0.159
Rosmarinic acid	TYR	0.158
Luteolin	GRB2	0.157
Calycosin	RERGL	0.157
Acacetin	MKNK1	0.156
Acacetin	MKNK2	0.156
Luteolin	RPTOR	0.156
Luteolin	CPSF3	0.155
Luteolin	CPSF3L	0.155
Luteolin	ENSG00000	0.155
Luteolin	GAB4	0.155
Luteolin	GIGYF1	0.155
Luteolin	GIGYF2	0.155
Luteolin	GTPBP10	0.155
Luteolin	GTPBP5	0.155
Luteolin	IST1	0.155
Luteolin	MTMR6	0.155
Luteolin	MTMR7	0.155
Luteolin	MTMR8	0.155
Luteolin	ATP5O	0.154
Luteolin	ENSG00000	0.154
Acacetin	CDK8	0.153
Luteolin	MRPS14	0.152
Acacetin	PPA2	0.152
Luteolin	SDHB	0.152
Luteolin	ZBTB8OS	0.152
Luteolin	MLH1	0.151
Luteolin	MLH3	0.151
Luteolin	PMS1	0.151
Luteolin	PMS2	0.151
Rosmarinic acid	ADAM17	0.15
Diosmetin	AHR	0.15
Luteolin	BAX	0.15
Calycosin	CCNB1	0.15
Calycosin	CCND3	0.15
Calycosin	CCNG1	0.15
Luteolin	CD74	0.15
Luteolin	CDK1	0.15

Calycosin	CDK7	0.15
Calycosin	CDKN1A	0.15
Luteolin	CDKN1A	0.15
Calycosin	CDKN1C	0.15
Calycosin	CDKN2D	0.15
Luteolin	CSF2	0.15
Rosmarinic acid	DNMT1	0.15
Luteolin	EGR1	0.15
Calycosin	EPO	0.15
Calycosin	ERP29	0.15
Luteolin	FBP1	0.15
Luteolin	GADD45B	0.15
Calycosin	HIF1A	0.15
Luteolin	IL4	0.15
Calycosin	KRT1	0.15
Calycosin	LASP1	0.15
Calycosin	MCM5	0.15
Calycosin	MCM6	0.15
Luteolin	MMP1	0.15
Luteolin	MPC2	0.15
Calycosin	NME1	0.15
Luteolin	NR1D1	0.15
Calycosin	PEBP1	0.15
Calycosin	PGAM1	0.15
Calycosin	PRDX1	0.15
Calycosin	PRDX2	0.15
Luteolin	PSCA	0.15
Luteolin	PTK6	0.15
Calycosin	RAD9A	0.15
Calycosin	RBX1	0.15
Luteolin	SFN	0.15
Calycosin	SKP1	0.15
Calycosin	SKP2	0.15
Calycosin	STIP1	0.15
Luteolin	TP53I3	0.15
Luteolin	TP63	0.15
Luteolin	TP73	0.15
Calycosin	UBE3A	0.15
Luteolin	VAV3	0.15
Luteolin	WISP2	0.15
