# Supplementary material for: Pharmacological Effects of Agastache rugosa against Gastritis Using a Network Pharmacology Approach
Source: Biomolecules. 2020 Sep 9;10(9):1298. doi: 10.3390/biom10091298 (PMC7565599; doi:10.3390/biom10091298)
Supplement: Supplementary file 1 [file biomolecules-10-01298-s001.zip › gastritis_supplementary_table3.pdf]

Supplemenatary table 3. Gatritis related genes in GeneCards DB

No.	Symbol	Description	Category	GIFtS	GC id	Score ▼
1	IL1B	Interleukin 1 Beta	Protein Coding	49	GC02M112829	41.89
2	IL1RN	Interleukin 1 Receptor Antagonist	Protein Coding	48	GC02P114906	35.98
3	CDH1	Cadherin 1	Protein Coding	50	GC16P068737	31.26
4	GAST	Gastrin	Protein Coding	40	GC17P041712	29.27
5	CXCL8	C-X-C Motif Chemokine Ligand 8	Protein Coding	42	GC04P073740	22.11
6	S100A8	S100 Calcium Binding Protein A8	Protein Coding	42	GC01M153391	21.97
7	TNF	Tumor Necrosis Factor	Protein Coding	52	GC06P033397	21.05
8	PGA3	Pepsinogen A3	Protein Coding	33	GC11P061203	20.06
9	PGA5	Pepsinogen A5	Protein Coding	34	GC11P061241	19.67
10	PGA4	Pepsinogen A4	Protein Coding	30	GC11P061222	19.59
11	PGC	Progastricsin	Protein Coding	42	GC06M041736	19.51
12	AIRE	Autoimmune Regulator	Protein Coding	45	GC21P044285	19.16
13	IL10	Interleukin 10	Protein Coding	47	GC01M206767	18.79
14	TFF2	Trefoil Factor 2	Protein Coding	41	GC21M042346	18.43
15	PTGS2	Prostaglandin-Endoperoxide Synthase 2	Protein Coding	48	GC01M186640	18.4
16	IL6	Interleukin 6	Protein Coding	49	GC07P022765	17.79
17	CHGA	Chromogranin A	Protein Coding	42	GC14P092923	17.58
18	TP53	Tumor Protein P53	Protein Coding	53	GC17M007661	17.27
19	ATP4A	ATPase H+/K+ Transporting Subunit Alpha	Protein Coding	42	GC19M041506	16.66
20	SST	Somatostatin	Protein Coding	42	GC03M187668	16.58
21	GHRL	Ghrelin And Obestatin Prepropeptide	Protein Coding	43	GC03M010285	16.56
22	TGFA	Transforming Growth Factor Alpha	Protein Coding	46	GC02M070447	15.96
23	KRAS	KRAS Proto-Oncogene, GTPase	Protein Coding	50	GC12M025204	15.7
24	NOS2	Nitric Oxide Synthase 2	Protein Coding	49	GC17M027756	15.31
25	CDX2	Caudal Type Homeobox 2	Protein Coding	43	GC13M027962	15.27
26	ODC1	Ornithine Decarboxylase 1	Protein Coding	47	GC02M010432	15.21
27	CAT	Catalase	Protein Coding	50	GC11P034460	14.97
28	ADH7	Alcohol Dehydrogenase 7 (Class IV), Mu Or Sigma Polypeptide	Protein Coding	44	GC04M099412	14.64
29	TLR4	Toll Like Receptor 4	Protein Coding	50	GC09P117704	14.5
30	MUC6	Mucin 6, Oligomeric Mucus/Gel-Forming	Protein Coding	38	GC11M001002	14.39
31	CYP2C19	Cytochrome P450 Family 2 Subfamily C Member 19	Protein Coding	47	GC10P094762	13.84
32	LRBA	LPS Responsive Beige-Like Anchor Protein	Protein Coding	41	GC04M150264	13.79
33	CCR6	C-C Motif Chemokine Receptor 6	Protein Coding	45	GC06P167111	13.62
34	FAS	Fas Cell Surface Death Receptor	Protein Coding	50	GC10P088969	13.58
35	FOXP3	Forkhead Box P3	Protein Coding	46	GC0XM049250	13.45
36	IL17A	Interleukin 17A	Protein Coding	42	GC06P052186	13.18
37	DEFB1	Defensin Beta 1	Protein Coding	39	GC08M006870	13.06
38	NOD1	Nucleotide Binding Oligomerization Domain Containing 1	Protein Coding	45	GC07M030424	13.04
39	CXCL1	C-X-C Motif Chemokine Ligand 1	Protein Coding	43	GC04P073869	12.86
40	DEFB4A	Defensin Beta 4A	Protein Coding	36	GC08P007895	12.7
41	CCL5	C-C Motif Chemokine Ligand 5	Protein Coding	43	GC17M035871	12.66
42	HRH2	Histamine Receptor H2	Protein Coding	43	GC05P175659	12.65
43	FASLG	Fas Ligand	Protein Coding	47	GC01P172628	12.64
44	MIF	Macrophage Migration Inhibitory Factor	Protein Coding	49	GC22P023894	12.62
45	PTGS1	Prostaglandin-Endoperoxide Synthase 1	Protein Coding	46	GC09P122370	12.25
46	CXCL5	C-X-C Motif Chemokine Ligand 5	Protein Coding	41	GC04M073995	12
47	DEFA5	Defensin Alpha 5	Protein Coding	38	GC08M007057	11.94
48	SCT	Secretin	Protein Coding	35	GC11M000628	11.86
49	CCKBR	Cholecystokinin B Receptor	Protein Coding	45	GC11P006259	11.72
50	AREG	Amphiregulin	Protein Coding	43	GC04P074445	11.52
51	EGF	Epidermal Growth Factor	Protein Coding	51	GC04P109912	11.48
52	ATP12A	ATPase H+/K+ Transporting Non-Gastric Alpha2 Subunit	Protein Coding	42	GC13P024680	11.45
53	ALB	Albumin	Protein Coding	50	GC04P073397	10.96
54	PTGES	Prostaglandin E Synthase	Protein Coding	41	GC09M129738	10.92
55	MALT1	MALT1 Paracaspase	Protein Coding	47	GC18P058671	10.71
56	HLA-DRB1	Major Histocompatibility Complex, Class II, DR Beta 1	Protein Coding	46	GC06M032578	10.59
57	RIPK1	Receptor Interacting Serine/Threonine Kinase 1	Protein Coding	49	GC06P003064	10.2
58	MEN1	Menin 1	Protein Coding	46	GC11M064803	9.96
59	RUNX3	RUNX Family Transcription Factor 3	Protein Coding	43	GC01M024899	9.83
60	STAT3	Signal Transducer And Activator Of Transcription 3	Protein Coding	53	GC17M042313	9.78
61	IFNGR1	Interferon Gamma Receptor 1	Protein Coding	49	GC06M137197	9.45
62	EGFR	Epidermal Growth Factor Receptor	Protein Coding	54	GC07P055019	9.42
63	SERPINA1	Serpin Family A Member 1	Protein Coding	49	GC14M094376	9.15
64	IL11	Interleukin 11	Protein Coding	41	GC19M055364	9.11
65	MET	MET Proto-Oncogene, Receptor Tyrosine Kinase	Protein Coding	54	GC07P116672	9.03
66	ERCC6	ERCC Excision Repair 6, Chromatin Remodeling Factor	Protein Coding	45	GC10M049454	8.79
67	CTSW	Cathepsin W	Protein Coding	39	GC11P065879	8.67
68	IL2	Interleukin 2	Protein Coding	46	GC04M122451	8.48
69	IL4	Interleukin 4	Protein Coding	46	GC05P132673	8.48
70	IFNG	Interferon Gamma	Protein Coding	48	GC12M068064	8.46
71	TFF3	Trefoil Factor 3	Protein Coding	41	GC21M042311	8.26
72	HGF	Hepatocyte Growth Factor	Protein Coding	52	GC07M081699	8.2
73	MIB2	Mindbomb E3 Ubiquitin Protein Ligase 2	Protein Coding	38	GC01P001614	7.94
74	PRKCD	Protein Kinase C Delta	Protein Coding	53	GC03P053156	7.88

75	RASGRP1	RAS Guanyl Releasing Protein 1	Protein Coding	46	GC15M038488	7.88
76	CASP10	Caspase 10	Protein Coding	48	GC02P201182	7.67
77	SKIV2L	Ski2 Like RNA Helicase	Protein Coding	43	GC06P031958	7.67
78	TTC37	Tetratricopeptide Repeat Domain 37	Protein Coding	39	GC05M095463	7.67
79	HIP1R	Huntingtin Interacting Protein 1 Related	Protein Coding	41	GC12P122834	7.49
80	BARX1	BARX Homeobox 1	Protein Coding	36	GC09M093951	7.49
81	SPAG11A	Sperm Associated Antigen 11A	Protein Coding	27	GC08P007847	7.49
82	NFKB1	Nuclear Factor Kappa B Subunit 1	Protein Coding	53	GC04P102501	7.16
83	IL2RA	Interleukin 2 Receptor Subunit Alpha	Protein Coding	50	GC10M006010	7.09
84	IL5	Interleukin 5	Protein Coding	45	GC05M132541	6.99
85	GZMB	Granzyme B	Protein Coding	45	GC14M024630	6.88
86	CBLIF	Cobalamin Binding Intrinsic Factor	Protein Coding	34	GC11M059829	6.78
87	CTNNA1	Catenin Alpha 1	Protein Coding	47	GC05P138613	6.53
88	PTPN11	Protein Tyrosine Phosphatase Non-Receptor Type 11	Protein Coding	53	GC12P112418	6.41
89	MAP3K6	Mitogen-Activated Protein Kinase Kinase Kinase 6	Protein Coding	42	GC01M027365	6.12
90	AGK	Acylglycerol Kinase	Protein Coding	41	GC07P141551	6.12
91	CD4	CD4 Molecule	Protein Coding	50	GC12P006786	6.03
92	TIMP1	TIMP Metallopeptidase Inhibitor 1	Protein Coding	45	GC0XP047583	5.83
93	CEL	Carboxyl Ester Lipase	Protein Coding	46	GC09P133061	5.73
94	MUC2	Mucin 2, Oligomeric Mucus/Gel-Forming	Protein Coding	39	GC11P001074	5.64
95	CCK	Cholecystokinin	Protein Coding	41	GC03M042274	5.56
96	MIR146A	MicroRNA 146a	RNA Gene	21	GC05P160485	5.54
97	GAD2	Glutamate Decarboxylase 2	Protein Coding	46	GC10P026216	5.5
98	IL13	Interleukin 13	Protein Coding	45	GC05P132656	5.37
99	JUN	Jun Proto-Oncogene, AP-1 Transcription Factor Subunit	Protein Coding	49	GC01M058780	5.31
100	MIR27A	MicroRNA 27a	RNA Gene	20	GC19M013987	5.31
101	CD8A	CD8a Molecule	Protein Coding	47	GC02M086784	5.3
102	PDIA3	Protein Disulfide Isomerase Family A Member 3	Protein Coding	45	GC15P043746	5.3
103	PLA2G5	Phospholipase A2 Group V	Protein Coding	43	GC01P020028	5.3
104	RFXANK	Regulatory Factor X Associated Ankyrin Containing Protein	Protein Coding	42	GC19P019192	5.3
105	GPSM2	G Protein Signaling Modulator 2	Protein Coding	41	GC01P108875	5.3
106	PES1	Pescadillo Ribosomal Biogenesis Factor 1	Protein Coding	38	GC22M030576	5.3
107	TTI1	TELO2 Interacting Protein 1	Protein Coding	36	GC20M037983	5.3
108	COPG2IT1	COPG2 Imprinted Transcript 1	RNA Gene	11	GC07P130543	5.3
109	AICDA	Activation Induced Cytidine Deaminase	Protein Coding	46	GC12M008602	5.24
110	HLA-DQA1	Major Histocompatibility Complex, Class II, DQ Alpha 1	Protein Coding	43	GC06P033442	5.2
111	FUT2	Fucosyltransferase 2	Protein Coding	43	GC19P048695	5.2
112	CD40LG	CD40 Ligand	Protein Coding	47	GC0XP136649	5.13
113	CCR3	C-C Motif Chemokine Receptor 3	Protein Coding	47	GC03P046227	5.06
114	ENTPD1	Ectonucleoside Triphosphate Diphosphohydrolase 1	Protein Coding	46	GC10P095711	4.97
115	BCL2L11	BCL2 Like 11	Protein Coding	45	GC02P111119	4.97
116	MIR142	MicroRNA 142	RNA Gene	17	GC17M058331	4.97
117	SPP1	Secreted Phosphoprotein 1	Protein Coding	46	GC04P087975	4.92
118	TNFSF13B	TNF Superfamily Member 13b	Protein Coding	45	GC13P108251	4.92
119	TIA1	TIA1 Cytotoxic Granule Associated RNA Binding Protein	Protein Coding	42	GC02M070209	4.92
120	SH2D1A	SH2 Domain Containing 1A	Protein Coding	46	GC0XP124227	4.86
121	MIRLET7A1	MicroRNA Let-7a-1	RNA Gene	19	GC09P094175	4.86
122	GAD1	Glutamate Decarboxylase 1	Protein Coding	51	GC02P170813	4.77
123	CASP3	Caspase 3	Protein Coding	50	GC04M184627	4.74
124	CTLA4	Cytotoxic T-Lymphocyte Associated Protein 4	Protein Coding	46	GC02P203867	4.74
125	HLA-A	Major Histocompatibility Complex, Class I, A	Protein Coding	46	GC06P033211	4.71
126	HLA-B	Major Histocompatibility Complex, Class I, B	Protein Coding	45	GC06M031289	4.71
127	HLA-C	Major Histocompatibility Complex, Class I, C	Protein Coding	44	GC06M031272	4.71
128	TGFB1	Transforming Growth Factor Beta 1	Protein Coding	52	GC19M041301	4.69
129	MMP7	Matrix Metallopeptidase 7	Protein Coding	48	GC11M102425	4.63
130	MPO	Myeloperoxidase	Protein Coding	50	GC17M058269	4.62
131	MUC5AC	Mucin 5AC, Oligomeric Mucus/Gel-Forming	Protein Coding	39	GC11P001151	4.54
132	CHUK	Component Of Inhibitor Of Nuclear Factor Kappa B Kinase Complex	Protein Coding	51	GC10M100188	4.53
133	NFKBIA	NFKB Inhibitor Alpha	Protein Coding	50	GC14M035401	4.53
134	PAK1	P21 (RAC1) Activated Kinase 1	Protein Coding	47	GC11M077321	4.53
135	COX5A	Cytochrome C Oxidase Subunit 5A	Protein Coding	43	GC15M074919	4.51
136	TH	Tyrosine Hydroxylase	Protein Coding	52	GC11M002163	4.33
137	DDC	Dopa Decarboxylase	Protein Coding	52	GC07M050458	4.33
138	ADAM10	ADAM Metallopeptidase Domain 10	Protein Coding	52	GC15M058588	4.33
139	CASR	Calcium Sensing Receptor	Protein Coding	50	GC03P122183	4.33
140	CYP11A1	Cytochrome P450 Family 11 Subfamily A Member 1	Protein Coding	49	GC15M074337	4.33
141	CYP17A1	Cytochrome P450 Family 17 Subfamily A Member 1	Protein Coding	49	GC10M102830	4.33
142	GPD2	Glycerol-3-Phosphate Dehydrogenase 2	Protein Coding	46	GC02P156435	4.33
143	PSAP	Prosaposin	Protein Coding	46	GC10M071816	4.33
144	VDAC1	Voltage Dependent Anion Channel 1	Protein Coding	46	GC05M133975	4.33
145	CD28	CD28 Molecule	Protein Coding	46	GC02P203706	4.33
146	CYP1A2	Cytochrome P450 Family 1 Subfamily A Member 2	Protein Coding	45	GC15P074748	4.33
147	CYP21A2	Cytochrome P450 Family 21 Subfamily A Member 2	Protein Coding	45	GC06P033435	4.33
148	TFAM	Transcription Factor A, Mitochondrial	Protein Coding	43	GC10P058385	4.33
149	TPH1	Tryptophan Hydroxylase 1	Protein Coding	43	GC11M018040	4.33
150	CCL11	C-C Motif Chemokine Ligand 11	Protein Coding	43	GC17P034285	4.33
151	EPX	Eosinophil Peroxidase	Protein Coding	42	GC17P058192	4.33

152	GNPTAB	N-Acetylglucosamine-1-Phosphate Transferase Subunits Alpha And Beta	Protein Coding	41	GC12M101745	4.33
153	TCN1	Transcobalamin 1	Protein Coding	40	GC11M060026	4.33
154	SIGLEC8	Sialic Acid Binding Ig Like Lectin 8	Protein Coding	38	GC19M051450	4.33
155	ICOSLG	Inducible T Cell Costimulator Ligand	Protein Coding	38	GC21M044222	4.33
156	CCL26	C-C Motif Chemokine Ligand 26	Protein Coding	38	GC07M075769	4.33
157	FIP1L1	Factor Interacting With PAPOLA And CPSF1	Protein Coding	38	GC04P053383	4.33
158	NDUFB1	NADH:Ubiquinone Oxidoreductase Subunit B1	Protein Coding	38	GC14M092116	4.33
159	RNASE2	Ribonuclease A Family Member 2	Protein Coding	38	GC14P021144	4.33
160	TRAPPC10	Trafficking Protein Particle Complex 10	Protein Coding	37	GC21P044012	4.33
161	CCL24	C-C Motif Chemokine Ligand 24	Protein Coding	36	GC07M075811	4.33
162	CDH26	Cadherin 26	Protein Coding	35	GC20P059958	4.33
163	NDUFC1	NADH:Ubiquinone Oxidoreductase Subunit C1	Protein Coding	35	GC04M139267	4.33
164	SQOR	Sulfide Quinone Oxidoreductase	Protein Coding	28	GC15P045632	4.33
165	H2AC18	H2A Clustered Histone 18	Protein Coding	26	GC01M149914	4.33
166	MIR21	MicroRNA 21	RNA Gene	21	GC17P059841	4.33
167	MLH1	MutL Homolog 1	Protein Coding	47	GC03P036993	4.29
168	MUC1	Mucin 1, Cell Surface Associated	Protein Coding	47	GC01M155185	4.28
169	HRAS	HRas Proto-Oncogene, GTPase	Protein Coding	52	GC11M000522	4.1
170	MIR29C	MicroRNA 29c	RNA Gene	16	GC01M207802	4.05
171	HSPD1	Heat Shock Protein Family D (Hsp60) Member 1	Protein Coding	47	GC02M197486	4.01
172	MIR181C	MicroRNA 181c	RNA Gene	18	GC19P013876	4.01
173	SOD1	Superoxide Dismutase 1	Protein Coding	52	GC21P031659	3.95
174	CDKN2A	Cyclin Dependent Kinase Inhibitor 2A	Protein Coding	51	GC09M021967	3.94
175	HDC	Histidine Decarboxylase	Protein Coding	45	GC15M050241	3.91
176	PTPN3	Protein Tyrosine Phosphatase Non-Receptor Type 3	Protein Coding	44	GC09M109375	3.91
177	BCL2	BCL2 Apoptosis Regulator	Protein Coding	52	GC18M063123	3.89
178	MMP9	Matrix Metallopeptidase 9	Protein Coding	53	GC20P046008	3.88
179	MIR24-1	MicroRNA 24-1	RNA Gene	17	GC09P095086	3.86
180	MTOR	Mechanistic Target Of Rapamycin Kinase	Protein Coding	53	GC01M011106	3.79
181	TG	Thyroglobulin	Protein Coding	43	GC08P132866	3.79
182	MIR150	MicroRNA 150	RNA Gene	20	GC19M049500	3.79
183	GSTP1	Glutathione S-Transferase Pi 1	Protein Coding	49	GC11P067583	3.75
184	TPO	Thyroid Peroxidase	Protein Coding	48	GC02P001374	3.71
185	F2	Coagulation Factor II, Thrombin	Protein Coding	48	GC11P046720	3.71
186	HSPG2	Heparan Sulfate Proteoglycan 2	Protein Coding	47	GC01M021822	3.71
187	CRP	C-Reactive Protein	Protein Coding	46	GC01M159715	3.71
188	ITGAE	Integrin Subunit Alpha E	Protein Coding	39	GC17M003722	3.71
189	MIR106A	MicroRNA 106a	RNA Gene	16	GC0XM134249	3.71
190	MGMT	O-6-Methylguanine-DNA Methyltransferase	Protein Coding	49	GC10P129467	3.54
191	MMP2	Matrix Metallopeptidase 2	Protein Coding	53	GC16P055390	3.51
192	ALPP	Alkaline Phosphatase, Placental	Protein Coding	45	GC02P232378	3.5
193	MUC4	Mucin 4, Cell Surface Associated	Protein Coding	39	GC03M195746	3.5
194	KRT7	Keratin 7	Protein Coding	42	GC12P052232	3.48
195	PCNA	Proliferating Cell Nuclear Antigen	Protein Coding	51	GC20M005114	3.44
196	HLA-DQB1	Major Histocompatibility Complex, Class II, DQ Beta 1	Protein Coding	44	GC06M032660	3.43
197	LEP	Leptin	Protein Coding	47	GC07P128241	3.42
198	CASP8	Caspase 8	Protein Coding	52	GC02P201233	3.42
199	CD69	CD69 Molecule	Protein Coding	41	GC12M013363	3.42
200	IL7R	Interleukin 7 Receptor	Protein Coding	47	GC05P035852	3.35
201	MIR9-1	MicroRNA 9-1	RNA Gene	19	GC01M156420	3.35
202	MIR200B	MicroRNA 200b	RNA Gene	18	GC01P001167	3.35
203	MIR25	MicroRNA 25	RNA Gene	17	GC07M100093	3.35
204	MIR30A	MicroRNA 30a	RNA Gene	17	GC06M071403	3.35
205	MIR129-1	MicroRNA 129-1	RNA Gene	16	GC07P128207	3.35
206	TNFRSF1A	TNF Receptor Superfamily Member 1A	Protein Coding	49	GC12M006328	3.26
207	TNFAIP3	TNF Alpha Induced Protein 3	Protein Coding	47	GC06P137866	3.26
208	CFLAR	CASP8 And FADD Like Apoptosis Regulator	Protein Coding	46	GC02P201117	3.26
209	STAT5A	Signal Transducer And Activator Of Transcription 5A	Protein Coding	46	GC17P042287	3.26
210	PTPN22	Protein Tyrosine Phosphatase Non-Receptor Type 22	Protein Coding	46	GC01M113813	3.26
211	MTR	5-Methyltetrahydrofolate-Homocysteine Methyltransferase	Protein Coding	46	GC01P236795	3.26
212	TCN2	Transcobalamin 2	Protein Coding	43	GC22P030606	3.26
213	SERPINA3	Serpin Family A Member 3	Protein Coding	43	GC14P094612	3.26
214	HNRNPC	Heterogeneous Nuclear Ribonucleoprotein C	Protein Coding	41	GC14M021210	3.26
215	MIR221	MicroRNA 221	RNA Gene	19	GC0XM045746	3.26
216	MIR92A1	MicroRNA 92a-1	RNA Gene	17	GC13P091420	3.26
217	SOD2	Superoxide Dismutase 2	Protein Coding	50	GC06M159669	3.16
218	MDM2	MDM2 Proto-Oncogene	Protein Coding	53	GC12P068808	3.09
219	STAT1	Signal Transducer And Activator Of Transcription 1	Protein Coding	53	GC02M190964	3.06
220	PTPRC	Protein Tyrosine Phosphatase Receptor Type C	Protein Coding	51	GC01P198607	3.06
221	JAK1	Janus Kinase 1	Protein Coding	50	GC01M064833	3.06
222	NRAS	NRAS Proto-Oncogene, GTPase	Protein Coding	50	GC01M114704	3.06
223	CBL	Cbl Proto-Oncogene	Protein Coding	50	GC11P119206	3.06
224	ALPL	Alkaline Phosphatase, Biomineralization Associated	Protein Coding	50	GC01P021508	3.06
225	BMPR2	Bone Morphogenetic Protein Receptor Type 2	Protein Coding	50	GC02P202376	3.06
226	STAT5B	Signal Transducer And Activator Of Transcription 5B	Protein Coding	49	GC17M042199	3.06
227	CD19	CD19 Molecule	Protein Coding	49	GC16P028943	3.06
228	ACVRL1	Activin A Receptor Like Type 1	Protein Coding	49	GC12P051906	3.06

229	TGM2	Transglutaminase 2	Protein Coding	48	GC20M038127	3.06
230	ACTA2	Actin Alpha 2, Smooth Muscle	Protein Coding	47	GC10M088935	3.06
231	FADD	Fas Associated Via Death Domain	Protein Coding	47	GC11P070203	3.06
232	HDAC9	Histone Deacetylase 9	Protein Coding	46	GC07P018086	3.06
233	PPT1	Palmitoyl-Protein Thioesterase 1	Protein Coding	46	GC01M040072	3.06
234	TBX21	T-Box Transcription Factor 21	Protein Coding	46	GC17P047733	3.06
235	SERPINH1	Serpin Family H Member 1	Protein Coding	46	GC11P075562	3.06
236	ARF1	ADP Ribosylation Factor 1	Protein Coding	46	GC01P228082	3.06
237	FST	Follistatin	Protein Coding	46	GC05P053480	3.06
238	ADAMTS13	ADAM Metallopeptidase With Thrombospondin Type 1 Motif 13	Protein Coding	46	GC09P133414	3.06
239	CR1	Complement C3b/C4b Receptor 1 (Knops Blood Group)	Protein Coding	45	GC01P207496	3.06
240	PRF1	Perforin 1	Protein Coding	45	GC10M070597	3.06
241	IL10RA	Interleukin 10 Receptor Subunit Alpha	Protein Coding	45	GC11P117987	3.06
242	SNRPN	Small Nuclear Ribonucleoprotein Polypeptide N	Protein Coding	45	GC15P024823	3.06
243	CD27	CD27 Molecule	Protein Coding	45	GC12P006608	3.06
244	MATK	Megakaryocyte-Associated Tyrosine Kinase	Protein Coding	45	GC19M003777	3.06
245	APOH	Apolipoprotein H	Protein Coding	44	GC17M066212	3.06
246	NLRP1	NLR Family Pyrin Domain Containing 1	Protein Coding	44	GC17M005499	3.06
247	TNFRSF18	TNF Receptor Superfamily Member 18	Protein Coding	43	GC01M001203	3.06
248	TOR1A	Torsin Family 1 Member A	Protein Coding	43	GC09M129812	3.06
249	UNC13D	Unc-13 Homolog D	Protein Coding	43	GC17M075827	3.06
250	PTPN5	Protein Tyrosine Phosphatase Non-Receptor Type 5	Protein Coding	43	GC11M018727	3.06
251	GYPC	Glycophorin C (Gerbich Blood Group)	Protein Coding	43	GC02P126655	3.06
252	PDE6D	Phosphodiesterase 6D	Protein Coding	43	GC02M231732	3.06
253	FOXP2	Forkhead Box P2	Protein Coding	43	GC07P114086	3.06
254	RIPK3	Receptor Interacting Serine/Threonine Kinase 3	Protein Coding	43	GC14M024336	3.06
255	ALPI	Alkaline Phosphatase, Intestinal	Protein Coding	43	GC02P232456	3.06
256	FAF1	Fas Associated Factor 1	Protein Coding	43	GC01M050439	3.06
257	TIMM8A	Translocase Of Inner Mitochondrial Membrane 8A	Protein Coding	42	GC0XM101345	3.06
258	TRIP11	Thyroid Hormone Receptor Interactor 11	Protein Coding	42	GC14M091965	3.06
259	NUP107	Nucleoporin 107	Protein Coding	42	GC12P068686	3.06
260	ARFGAP1	ADP Ribosylation Factor GTPase Activating Protein 1	Protein Coding	42	GC20P063272	3.06
261	DOCK8	Dedicator Of Cytokinesis 8	Protein Coding	42	GC09P000214	3.06
262	MLKL	Mixed Lineage Kinase Domain Like Pseudokinase	Protein Coding	41	GC16M074672	3.06
263	NT5C	5', 3'-Nucleotidase, Cytosolic	Protein Coding	41	GC17M075130	3.06
264	NUP133	Nucleoporin 133	Protein Coding	41	GC01M229441	3.06
265	CD68	CD68 Molecule	Protein Coding	40	GC17P007579	3.06
266	NUP85	Nucleoporin 85	Protein Coding	40	GC17P075205	3.06
267	ARFGAP3	ADP Ribosylation Factor GTPase Activating Protein 3	Protein Coding	40	GC22M042796	3.06
268	CLPS	Colipase	Protein Coding	40	GC06M041294	3.06
269	HNRNPDL	Heterogeneous Nuclear Ribonucleoprotein D Like	Protein Coding	39	GC04M082422	3.06
270	NUP160	Nucleoporin 160	Protein Coding	39	GC11M059719	3.06
271	SMTN	Smoothelin	Protein Coding	39	GC22P031066	3.06
272	SNRNP70	Small Nuclear Ribonucleoprotein U1 Subunit 70	Protein Coding	39	GC19P049085	3.06
273	SNRPA	Small Nuclear Ribonucleoprotein Polypeptide A	Protein Coding	39	GC19P040750	3.06
274	IFI30	IFI30 Lysosomal Thiol Reductase	Protein Coding	39	GC19P018173	3.06
275	PIR	Pirin	Protein Coding	39	GC0XM015402	3.06
276	LSM2	LSM2 Homolog, U6 Small Nuclear RNA And MRNA Degradation Associated	Protein Coding	39	GC06M032380	3.06
277	MAGT1	Magnesium Transporter 1	Protein Coding	39	GC0XM077826	3.06
278	CHMP2A	Charged Multivesicular Body Protein 2A	Protein Coding	38	GC19M058551	3.06
279	RAB3IL1	RAB3A Interacting Protein Like 1	Protein Coding	37	GC11M061897	3.06
280	LAG3	Lymphocyte Activating 3	Protein Coding	37	GC12P006857	3.06
281	NUP37	Nucleoporin 37	Protein Coding	37	GC12M102073	3.06
282	C1QTNF6	C1q And TNF Related 6	Protein Coding	37	GC22M037180	3.06
283	DDX21	DExD-Box Helicase 21	Protein Coding	37	GC10P068956	3.06
284	ACKR1	Atypical Chemokine Receptor 1 (Duffy Blood Group)	Protein Coding	37	GC01P159203	3.06
285	PRSS27	Serine Protease 27	Protein Coding	36	GC16M002713	3.06
286	PAM16	Presequence Translocase Associated Motor 16	Protein Coding	36	GC16M004332	3.06
287	SNRPD3	Small Nuclear Ribonucleoprotein D3 Polypeptide	Protein Coding	36	GC22P024555	3.06
288	ARFGAP2	ADP Ribosylation Factor GTPase Activating Protein 2	Protein Coding	36	GC11M059684	3.06
289	AHCTF1	AT-Hook Containing Transcription Factor 1	Protein Coding	36	GC01M246840	3.06
290	ANKRD49	Ankyrin Repeat Domain 49	Protein Coding	36	GC11P094493	3.06
291	MMD	Monocyte To Macrophage Differentiation Associated	Protein Coding	35	GC17M055392	3.06
292	VILL	Villin Like	Protein Coding	35	GC03P037989	3.06
293	RNPC3	RNA Binding Region (RNP1, RRM) Containing 3	Protein Coding	35	GC01P103525	3.06
294	MUC3A	Mucin 3A, Cell Surface Associated	Protein Coding	35	GC07P100949	3.06
295	NUP43	Nucleoporin 43	Protein Coding	34	GC06M149724	3.06
296	NUDT10	Nudix Hydrolase 10	Protein Coding	34	GC0XP051332	3.06
297	MAS1L	MAS1 Proto-Oncogene Like, G Protein-Coupled Receptor	Protein Coding	34	GC06M029519	3.06
298	MT-CO1	Mitochondrially Encoded Cytochrome C Oxidase I	Protein Coding	34	GCMTP005906	3.06
299	ZNF740	Zinc Finger Protein 740	Protein Coding	33	GC12P053180	3.06
300	MT-CYB	Mitochondrially Encoded Cytochrome B	Protein Coding	32	GCMTP014749	3.06
301	ALPG	Alkaline Phosphatase, Germ Cell	Protein Coding	32	GC02P232407	3.06
302	GET1	Guided Entry Of Tail-Anchored Proteins Factor 1	Protein Coding	26	GC21P039377	3.06
303	MIR34A	MicroRNA 34a	RNA Gene	20	GC01M009151	3.06
304	MIR16-1	MicroRNA 16-1	RNA Gene	20	GC13M050048	3.06
305	MIR143	MicroRNA 143	RNA Gene	20	GC05P149410	3.06

306	MIR145	MicroRNA 145	RNA Gene	20	GC05P149430	3.06
307	MIR126	MicroRNA 126	RNA Gene	20	GC09P136670	3.06
308	MIR34C	MicroRNA 34c	RNA Gene	19	GC11P111579	3.06
309	MIR192	MicroRNA 192	RNA Gene	19	GC11M064891	3.06
310	MIR99A	MicroRNA 99a	RNA Gene	19	GC21P016539	3.06
311	MIR10B	MicroRNA 10b	RNA Gene	19	GC02P176150	3.06
312	MIR17	MicroRNA 17	RNA Gene	19	GC13P091350	3.06
313	MIR122	MicroRNA 122	RNA Gene	19	GC18P058451	3.06
314	MIR124-1	MicroRNA 124-1	RNA Gene	19	GC08M009903	3.06
315	MIR29A	MicroRNA 29a	RNA Gene	19	GC07M130876	3.06
316	MIR29B1	MicroRNA 29b-1	RNA Gene	19	GC07M130877	3.06
317	LINC01554	Long Intergenic Non-Protein Coding RNA 1554	RNA Gene	19	GC05P095838	3.06
318	MIR185	MicroRNA 185	RNA Gene	19	GC22P020034	3.06
319	MIR200A	MicroRNA 200a	RNA Gene	19	GC01P001296	3.06
320	MIR140	MicroRNA 140	RNA Gene	19	GC16P069934	3.06
321	MIR125A	MicroRNA 125a	RNA Gene	19	GC19P051723	3.06
322	MIR125B1	MicroRNA 125b-1	RNA Gene	19	GC11M122100	3.06
323	MIR149	MicroRNA 149	RNA Gene	19	GC02P240456	3.06
324	MIR30E	MicroRNA 30e	RNA Gene	19	GC01P040754	3.06
325	MIR93	MicroRNA 93	RNA Gene	18	GC07M100246	3.06
326	MIR373	MicroRNA 373	RNA Gene	18	GC19P054121	3.06
327	MIR23A	MicroRNA 23a	RNA Gene	18	GC19M013988	3.06
328	MIR23B	MicroRNA 23b	RNA Gene	18	GC09P095085	3.06
329	MIR127	MicroRNA 127	RNA Gene	18	GC14P104252	3.06
330	MIR483	MicroRNA 483	RNA Gene	17	GC11M002178	3.06
331	MIR181A1	MicroRNA 181a-1	RNA Gene	17	GC01M198860	3.06
332	MIR101-1	MicroRNA 101-1	RNA Gene	17	GC01M065058	3.06
333	MIR182	MicroRNA 182	RNA Gene	17	GC07M129770	3.06
334	MIR139	MicroRNA 139	RNA Gene	17	GC11M072615	3.06
335	MIR146B	MicroRNA 146b	RNA Gene	17	GC10P102436	3.06
336	MIR212	MicroRNA 212	RNA Gene	17	GC17M002050	3.06
337	MIR214	MicroRNA 214	RNA Gene	17	GC01M172229	3.06
338	MIR22	MicroRNA 22	RNA Gene	17	GC17M001713	3.06
339	MIR195	MicroRNA 195	RNA Gene	17	GC17M007018	3.06
340	MIR196A1	MicroRNA 196a-1	RNA Gene	17	GC17M048632	3.06
341	MIR196B	MicroRNA 196b	RNA Gene	17	GC07M027224	3.06
342	MIR199A1	MicroRNA 199a-1	RNA Gene	17	GC19M010792	3.06
343	MIR31	MicroRNA 31	RNA Gene	17	GC09M021513	3.06
344	MIR331	MicroRNA 331	RNA Gene	16	GC12P095308	3.06
345	MIR335	MicroRNA 335	RNA Gene	16	GC07P130496	3.06
346	MIR574	MicroRNA 574	RNA Gene	16	GC04P038872	3.06
347	MIR590	MicroRNA 590	RNA Gene	16	GC07P074191	3.06
348	MIR423	MicroRNA 423	RNA Gene	16	GC17P030117	3.06
349	MIR103A1	MicroRNA 103a-1	RNA Gene	16	GC05M168560	3.06
350	MIR130B	MicroRNA 130b	RNA Gene	16	GC22P023989	3.06
351	MIR15B	MicroRNA 15b	RNA Gene	16	GC03P160404	3.06
352	MIR455	MicroRNA 455	RNA Gene	16	GC09P114209	3.06
353	MIR186	MicroRNA 186	RNA Gene	16	GC01M071067	3.06
354	MIR18A	MicroRNA 18a	RNA Gene	16	GC13P091422	3.06
355	MIR20A	MicroRNA 20a	RNA Gene	16	GC13P091427	3.06
356	MIR151A	MicroRNA 151a	RNA Gene	16	GC08M140733	3.06
357	MIR193A	MicroRNA 193a	RNA Gene	16	GC17P031559	3.06
358	MIR675	MicroRNA 675	RNA Gene	15	GC11M001997	3.06
359	MIR338	MicroRNA 338	RNA Gene	15	GC17M081126	3.06
360	MIR486-1	MicroRNA 486-1	RNA Gene	15	GC08M041660	3.06
361	MIR371A	MicroRNA 371a	RNA Gene	15	GC19P053787	3.06
362	MIR361	MicroRNA 361	RNA Gene	15	GC0XM085903	3.06
363	MIR144	MicroRNA 144	RNA Gene	14	GC17M029703	3.06
364	MIR15A	MicroRNA 15a	RNA Gene	13	GC13M050049	3.06
365	MT-TF	Mitochondrially Encoded TRNA-Phe (UUU/C)	RNA Gene	13	GCMTP000580	3.06
366	MT-TH	Mitochondrially Encoded TRNA-His (CAU/C)	RNA Gene	12	GCMTP012140	3.06
367	LOC1019273	Uncharacterized LOC101927322	RNA Gene	11	GC18M058670	3.06
368	CDR1-AS	CDR1 Antisense RNA	RNA Gene	7	GC0XU902169	3.06
369	LEPQTL1	Leptin, Serum Levels Of	Genetic Locus	4	GC02U903086	3.06
370	MBL2	Mannose Binding Lectin 2	Protein Coding	47	GC10M052760	2.99
371	ERBB2	Erb-B2 Receptor Tyrosine Kinase 2	Protein Coding	54	GC17P039687	2.97
372	PPARG	Peroxisome Proliferator Activated Receptor Gamma	Protein Coding	53	GC03P012287	2.97
373	COL14A1	Collagen Type XIV Alpha 1 Chain	Protein Coding	42	GC08P120060	2.94
374	CA10	Carbonic Anhydrase 10	Protein Coding	39	GC17M051630	2.94
375	MRPL13	Mitochondrial Ribosomal Protein L13	Protein Coding	38	GC08M120377	2.94
376	MTBP	MDM2 Binding Protein	Protein Coding	34	GC08P120426	2.94
377	LY86-AS1	LY86 Antisense RNA 1	RNA Gene	16	GC06M006349	2.94
378	LTF	Lactotransferrin	Protein Coding	43	GC03M046435	2.92
379	OGG1	8-Oxoguanine DNA Glycosylase	Protein Coding	47	GC03P009751	2.89
380	MTHFR	Methylenetetrahydrofolate Reductase	Protein Coding	47	GC01M011785	2.84
381	IL1A	Interleukin 1 Alpha	Protein Coding	44	GC02M112773	2.81
382	XRCC1	X-Ray Repair Cross Complementing 1	Protein Coding	43	GC19M043543	2.81

383	CTNNB1	Catenin Beta 1	Protein Coding	53	GC03P041236	2.77
384	VEGFA	Vascular Endothelial Growth Factor A	Protein Coding	49	GC06P043770	2.74
385	LTA	Lymphotoxin Alpha	Protein Coding	42	GC06P033395	2.74
386	ERCC8	ERCC Excision Repair 8, CSA Ubiquitin Ligase Complex Subunit	Protein Coding	40	GC05M060873	2.7
387	CDKN1B	Cyclin Dependent Kinase Inhibitor 1B	Protein Coding	49	GC12P012716	2.67
388	GSTM1	Glutathione S-Transferase Mu 1	Protein Coding	41	GC01P109687	2.67
389	GSTT1	Glutathione S-Transferase Theta 1	Protein Coding	33	GC22Mi00270	2.67
390	CCL2	C-C Motif Chemokine Ligand 2	Protein Coding	49	GC17P034255	2.62
391	PSCA	Prostate Stem Cell Antigen	Protein Coding	39	GC08P142670	2.6
392	TXN	Thioredoxin	Protein Coding	46	GC09M110243	2.59
393	ABCB1	ATP Binding Cassette Subfamily B Member 1	Protein Coding	51	GC07M087504	2.58
394	ICAM1	Intercellular Adhesion Molecule 1	Protein Coding	50	GC19P010270	2.58
395	TLR1	Toll Like Receptor 1	Protein Coding	47	GC04M038797	2.55
396	TLR10	Toll Like Receptor 10	Protein Coding	41	GC04M038773	2.55
397	CYP2E1	Cytochrome P450 Family 2 Subfamily E Member 1	Protein Coding	45	GC10P133520	2.52
398	CD44	CD44 Molecule (Indian Blood Group)	Protein Coding	47	GC11P035139	2.51
399	IL23A	Interleukin 23 Subunit Alpha	Protein Coding	39	GC12P056335	2.49
400	CCL20	C-C Motif Chemokine Ligand 20	Protein Coding	44	GC02P227813	2.46
401	NOD2	Nucleotide Binding Oligomerization Domain Containing 2	Protein Coding	48	GC16P050693	2.46
402	AQP3	Aquaporin 3 (Gill Blood Group)	Protein Coding	47	GC09M033431	2.41
403	AQP4	Aquaporin 4	Protein Coding	44	GC18M026852	2.41
404	MIP	Major Intrinsic Protein Of Lens Fiber	Protein Coding	41	GC12M056449	2.41
405	TP73	Tumor Protein P73	Protein Coding	46	GC01P003652	2.39
406	KRT18	Keratin 18	Protein Coding	49	GC12P052948	2.38
407	TFF1	Trefoil Factor 1	Protein Coding	45	GC21M042362	2.37
408	KCNE2	Potassium Voltage-Gated Channel Subfamily E Regulatory Subunit 2	Protein Coding	41	GC21P034364	2.34
409	BIRC5	Baculoviral IAP Repeat Containing 5	Protein Coding	46	GC17P078214	2.32
410	THBS1	Thrombospondin 1	Protein Coding	45	GC15P039581	2.32
411	TIMP3	TIMP Metallopeptidase Inhibitor 3	Protein Coding	45	GC22P032800	2.32
412	CMA1	Chymase 1	Protein Coding	43	GC14M024506	2.32
413	MIR155	MicroRNA 155	RNA Gene	16	GC21P025573	2.28
414	KRT20	Keratin 20	Protein Coding	41	GC17M040875	2.28
415	PARP1	Poly(ADP-Ribose) Polymerase 1	Protein Coding	49	GC01M226360	2.23
416	MSH2	MutS Homolog 2	Protein Coding	48	GC02P047402	2.23
417	CD59	CD59 Molecule (CD59 Blood Group)	Protein Coding	46	GC11M033704	2.23
418	ALDH2	Aldehyde Dehydrogenase 2 Family Member	Protein Coding	50	GC12P111766	2.22
419	CD55	CD55 Molecule (Cromer Blood Group)	Protein Coding	47	GC01P207321	2.22
420	ACE	Angiotensin I Converting Enzyme	Protein Coding	49	GC17P063477	2.19
421	XDH	Xanthine Dehydrogenase	Protein Coding	47	GC02M031294	2.19
422	RASSF1	Ras Association Domain Family Member 1	Protein Coding	43	GC03M050329	2.19
423	TRPV1	Transient Receptor Potential Cation Channel Subfamily V Member 1	Protein Coding	46	GC17M003565	2.18
424	PDX1	Pancreatic And Duodenal Homeobox 1	Protein Coding	46	GC13P027921	2.18
425	KRT8	Keratin 8	Protein Coding	47	GC12M052897	2.16
426	CCR5	C-C Motif Chemokine Receptor 5 (Gene/Pseudogene)	Protein Coding	47	GC03P046384	2.14
427	CXCR3	C-X-C Motif Chemokine Receptor 3	Protein Coding	44	GC0XM071615	2.14
428	SKP2	S-Phase Kinase Associated Protein 2	Protein Coding	44	GC05P036103	2.14
429	KRT19	Keratin 19	Protein Coding	45	GC17M041523	2.13
430	ADIPOQ	Adiponectin, C1Q And Collagen Domain Containing	Protein Coding	45	GC03P186842	2.13
431	ADA	Adenosine Deaminase	Protein Coding	50	GC20M044620	2.13
432	CD46	CD46 Molecule	Protein Coding	46	GC01P207752	2.09
433	SELE	Selectin E	Protein Coding	44	GC01M169722	2.09
434	HSPA4	Heat Shock Protein Family A (Hsp70) Member 4	Protein Coding	42	GC05P133051	2.09
435	IL33	Interleukin 33	Protein Coding	39	GC09P006206	2.09
436	IL2RB	Interleukin 2 Receptor Subunit Beta	Protein Coding	48	GC22M037125	2.07
437	HBEGF	Heparin Binding EGF Like Growth Factor	Protein Coding	43	GC05M140332	2.07
438	VIP	Vasoactive Intestinal Peptide	Protein Coding	45	GC06P152750	2.05
439	THBD	Thrombomodulin	Protein Coding	44	GC20M023026	2.05
440	IL21	Interleukin 21	Protein Coding	43	GC04M122612	2.05
441	CCL21	C-C Motif Chemokine Ligand 21	Protein Coding	43	GC09M034709	2.05
442	CALCA	Calcitonin Related Polypeptide Alpha	Protein Coding	43	GC11M014945	2.03
443	XRCC3	X-Ray Repair Cross Complementing 3	Protein Coding	41	GC14M103697	2.03
444	SLC39A11	Solute Carrier Family 39 Member 11	Protein Coding	37	GC17M072645	2.03
445	KRT1	Keratin 1	Protein Coding	45	GC12M052674	1.99
446	ANXA1	Annexin A1	Protein Coding	49	GC09P073151	1.99
447	ITGB7	Integrin Subunit Beta 7	Protein Coding	44	GC12M053191	1.99
448	FUT3	Fucosyltransferase 3 (Lewis Blood Group)	Protein Coding	42	GC19M005843	1.99
449	SLPI	Secretory Leukocyte Peptidase Inhibitor	Protein Coding	39	GC20M045252	1.99
450	KIT	KIT Proto-Oncogene, Receptor Tyrosine Kinase	Protein Coding	53	GC04P054657	1.95
451	ERBB3	Erb-B2 Receptor Tyrosine Kinase 3	Protein Coding	53	GC12P056086	1.95
452	TP53INP1	Tumor Protein P53 Inducible Nuclear Protein 1	Protein Coding	38	GC08M094925	1.95
453	TP53AIP1	Tumor Protein P53 Regulated Apoptosis Inducing Protein 1	Protein Coding	36	GC11M128934	1.95
454	MIR203A	MicroRNA 203a	RNA Gene	17	GC14P104331	1.95
455	NFE2L2	Nuclear Factor, Erythroid 2 Like 2	Protein Coding	47	GC02M177227	1.92
456	NT5E	5'-Nucleotidase Ecto	Protein Coding	50	GC06P085449	1.91
457	MMP8	Matrix Metallopeptidase 8	Protein Coding	47	GC11M102617	1.91
458	PDHX	Pyruvate Dehydrogenase Complex Component X	Protein Coding	47	GC11P034894	1.91
459	ITGAL	Integrin Subunit Alpha L	Protein Coding	46	GC16P030472	1.91

460	ITGAM	Integrin Subunit Alpha M	Protein Coding	46	GC16P031420	1.91
461	CCR4	C-C Motif Chemokine Receptor 4	Protein Coding	45	GC03P032951	1.91
462	CANT1	Calcium Activated Nucleotidase 1	Protein Coding	43	GC17M078992	1.91
463	CDH13	Cadherin 13	Protein Coding	42	GC16P082626	1.91
464	L1TD1	LINE1 Type Transposase Domain Containing 1	Protein Coding	34	GC01P062194	1.91
465	MIR429	MicroRNA 429	RNA Gene	18	GC01P001297	1.91
466	BRAF	B-Raf Proto-Oncogene, Serine/Threonine Kinase	Protein Coding	54	GC07M140719	1.86
467	INS	Insulin	Protein Coding	48	GC11M002159	1.86
468	ELANE	Elastase, Neutrophil Expressed	Protein Coding	47	GC19P000854	1.86
469	GH1	Growth Hormone 1	Protein Coding	45	GC17M063917	1.86
470	APEX1	Apurinic/Apyrimidinic Endodeoxyribonuclease 1	Protein Coding	45	GC14P020455	1.86
471	CCR7	C-C Motif Chemokine Receptor 7	Protein Coding	45	GC17M040556	1.86
472	IL12A	Interleukin 12A	Protein Coding	44	GC03P159988	1.86
473	FLNC	Filamin C	Protein Coding	44	GC07P128830	1.86
474	HAND1	Heart And Neural Crest Derivatives Expressed 1	Protein Coding	41	GC05M154450	1.86
475	CXCL2	C-X-C Motif Chemokine Ligand 2	Protein Coding	41	GC04M074097	1.86
476	BGLAP	Bone Gamma-Carboxyglutamate Protein	Protein Coding	40	GC01P156242	1.86
477	CCN2	Cellular Communication Network Factor 2	Protein Coding	39	GC06M131948	1.86
478	RNASE7	Ribonuclease A Family Member 7	Protein Coding	35	GC14P021042	1.86
479	TLR2	Toll Like Receptor 2	Protein Coding	51	GC04P153684	1.81
480	BCL2L1	BCL2 Like 1	Protein Coding	47	GC20M031664	1.81
481	ARSA	Arylsulfatase A	Protein Coding	47	GC22M050622	1.81
482	F13A1	Coagulation Factor XIII A Chain	Protein Coding	45	GC06M006144	1.81
483	NKX2-1	NK2 Homeobox 1	Protein Coding	45	GC14M036516	1.81
484	ADORA2A	Adenosine A2a Receptor	Protein Coding	45	GC22P024417	1.81
485	S100A1	S100 Calcium Binding Protein A1	Protein Coding	42	GC01P153627	1.81
486	CCL19	C-C Motif Chemokine Ligand 19	Protein Coding	42	GC09M034692	1.81
487	RNASE3	Ribonuclease A Family Member 3	Protein Coding	40	GC14P020891	1.81
488	ABO	ABO, Alpha 1-3-N-Acetylgalactosaminyltransferase And Alpha 1-3-Galactosyltransferase	Protein Coding	38	GC09M133250	1.81
489	GAL3ST1	Galactose-3-O-Sulfotransferase 1	Protein Coding	36	GC22M030554	1.81
490	FOXJ1	Forkhead Box J1	Protein Coding	35	GC17M076136	1.81
491	RNF180	Ring Finger Protein 180	Protein Coding	32	GC05P064165	1.81
492	HSPB1	Heat Shock Protein Family B (Small) Member 1	Protein Coding	51	GC07P076302	1.74
493	TNFSF10	TNF Superfamily Member 10	Protein Coding	46	GC03M172505	1.74
494	LGALS3	Galectin 3	Protein Coding	45	GC14P055124	1.74
495	BAK1	BCL2 Antagonist/Killer 1	Protein Coding	45	GC06M033572	1.74
496	LGALS1	Galectin 1	Protein Coding	43	GC22P037675	1.74
497	NEU1	Neuraminidase 1	Protein Coding	43	GC06M031857	1.74
498	AMH	Anti-Mullerian Hormone	Protein Coding	43	GC19P002251	1.74
499	CTTN	Cortactin	Protein Coding	42	GC11P070398	1.74
500	LY96	Lymphocyte Antigen 96	Protein Coding	42	GC08P073991	1.74
501	CCL28	C-C Motif Chemokine Ligand 28	Protein Coding	40	GC05M043356	1.74
502	LGALS9	Galectin 9	Protein Coding	39	GC17P027629	1.74
503	SAPCD2	Suppressor APC Domain Containing 2	Protein Coding	31	GC09M137062	1.74
504	IGHE	Immunoglobulin Heavy Constant Epsilon	Protein Coding	28	GC14M105826	1.74
505	TERT	Telomerase Reverse Transcriptase	Protein Coding	52	GC05M001253	1.66
506	APOA1	Apolipoprotein A1	Protein Coding	49	GC11M116835	1.66
507	ALOX5	Arachidonate 5-Lipoxygenase	Protein Coding	48	GC10P045374	1.66
508	PRKAA1	Protein Kinase AMP-Activated Catalytic Subunit Alpha 1	Protein Coding	47	GC05M040759	1.66
509	GHR	Growth Hormone Receptor	Protein Coding	47	GC05P042429	1.66
510	ERCC2	ERCC Excision Repair 2, TFIIH Core Complex Helicase Subunit	Protein Coding	46	GC19M045349	1.66
511	PTGES3	Prostaglandin E Synthase 3	Protein Coding	45	GC12M056667	1.66
512	XPC	XPC Complex Subunit, DNA Damage Recognition And Repair Factor	Protein Coding	45	GC03M015413	1.66
513	ERCC4	ERCC Excision Repair 4, Endonuclease Catalytic Subunit	Protein Coding	45	GC16P013920	1.66
514	ERCC5	ERCC Excision Repair 5, Endonuclease	Protein Coding	45	GC13P102808	1.66
515	DDB2	Damage Specific DNA Binding Protein 2	Protein Coding	45	GC11P047237	1.66
516	LTB4R	Leukotriene B4 Receptor	Protein Coding	44	GC14P024311	1.66
517	GAB1	GRB2 Associated Binding Protein 1	Protein Coding	44	GC04P143336	1.66
518	TNFRSF10D	TNF Receptor Superfamily Member 10d	Protein Coding	43	GC08M023135	1.66
519	MUC5B	Mucin 5B, Oligomeric Mucus/Gel-Forming	Protein Coding	42	GC11P001244	1.66
520	CYSLTR1	Cysteinyl Leukotriene Receptor 1	Protein Coding	42	GC0XM078271	1.66
521	NDUFS5	NADH:Ubiquinone Oxidoreductase Subunit S5	Protein Coding	41	GC01P039026	1.66
522	BLVRB	Biliverdin Reductase B	Protein Coding	40	GC19M040447	1.66
523	KIR3DL1	Killer Cell Immunoglobulin Like Receptor, Three Ig Domains And Long Cytoplasmic Tail	Protein Coding	39	GC19P055293	1.66
524	CCL25	C-C Motif Chemokine Ligand 25	Protein Coding	39	GC19P008117	1.66
525	KIR2DL4	Killer Cell Immunoglobulin Like Receptor, Two Ig Domains And Long Cytoplasmic Tail 4	Protein Coding	38	GC19P055292	1.66
526	KIR3DL2	Killer Cell Immunoglobulin Like Receptor, Three Ig Domains And Long Cytoplasmic Tail	Protein Coding	38	GC19P055295	1.66
527	MADCAM1	Mucosal Vascular Addressin Cell Adhesion Molecule 1	Protein Coding	38	GC19P000499	1.66
528	SSPN	Sarcospan	Protein Coding	36	GC12P026118	1.66
529	KIR2DL3	Killer Cell Immunoglobulin Like Receptor, Two Ig Domains And Long Cytoplasmic Tail 3	Protein Coding	36	GC19P055318	1.66
530	KIR2DL1	Killer Cell Immunoglobulin Like Receptor, Two Ig Domains And Long Cytoplasmic Tail 1	Protein Coding	35	GC19P055289	1.66
531	KIR3DL3	Killer Cell Immunoglobulin Like Receptor, Three Ig Domains And Long Cytoplasmic Tail	Protein Coding	35	GC19P055319	1.66
532	KIR2DS4	Killer Cell Immunoglobulin Like Receptor, Two Ig Domains And Short Cytoplasmic Tail 4	Protein Coding	32	GC19P055294	1.66
533	KIR2DL2	Killer Cell Immunoglobulin Like Receptor, Two Ig Domains And Long Cytoplasmic Tail 2	Protein Coding	24	GC19MP00144	1.66
534	KIR2DS2	Killer Cell Immunoglobulin Like Receptor, Two Ig Domains And Short Cytoplasmic Tail 2	Protein Coding	24	GC19MR00122	1.66
535	KIR2DS3	Killer Cell Immunoglobulin Like Receptor, Two Ig Domains And Short Cytoplasmic Tail 3	Protein Coding	24	GC19ME00037	1.66
536	KIR2DS5	Killer Cell Immunoglobulin Like Receptor, Two Ig Domains And Short Cytoplasmic Tail 5	Protein Coding	24	GC19MR00030	1.66

537	KIR2DL5A	Killer Cell Immunoglobulin Like Receptor, Two Ig Domains And Long Cytoplasmic Tail 5	Protein Coding	23	GC19MR00046	1.66
538	KIR2DS1	Killer Cell Immunoglobulin Like Receptor, Two Ig Domains And Short Cytoplasmic Tail 1	Protein Coding	23	GC19MR00013	1.66
539	KIR3DS1	Killer Cell Immunoglobulin Like Receptor, Three Ig Domains And Short Cytoplasmic Tail	Protein Coding	21	GC19MR00058	1.66
540	KIR3DP1	Killer Cell Immunoglobulin Like Receptor, Three Ig Domains Pseudogene 1	Pseudogene	15	GC19P055291	1.66
541	KIR2DP1	Killer Cell Immunoglobulin Like Receptor, Two Ig Domains Pseudogene 1	Pseudogene	10	GC19P055258	1.66
542	IGES	Immunoglobulin E Concentration, Serum	Genetic Locus	5	GC05U990033	1.66
543	APC	APC Regulator Of WNT Signaling Pathway	Protein Coding	48	GC05P112707	1.64
544	TYK2	Tyrosine Kinase 2	Protein Coding	53	GC19M010350	1.64
545	SHH	Sonic Hedgehog Signaling Molecule	Protein Coding	49	GC07M155799	1.63
546	CD14	CD14 Molecule	Protein Coding	45	GC05M140631	1.6
547	MYC	MYC Proto-Oncogene, BHLH Transcription Factor	Protein Coding	52	GC08P127735	1.57
548	TLR5	Toll Like Receptor 5	Protein Coding	47	GC01M223109	1.54
549	TLR9	Toll Like Receptor 9	Protein Coding	46	GC03M052222	1.54
550	LYZ	Lysozyme	Protein Coding	47	GC12P069348	1.45
551	NUCB2	Nucleobindin 2	Protein Coding	39	GC11P017218	1.45
552	GABPA	GA Binding Protein Transcription Factor Subunit Alpha	Protein Coding	39	GC21P025734	1.45
553	IL17C	Interleukin 17C	Protein Coding	38	GC16P088638	1.45
554	GRP	Gastrin Releasing Peptide	Protein Coding	41	GC18P059220	1.43
555	S1PR5	Sphingosine-1-Phosphate Receptor 5	Protein Coding	45	GC19M010512	1.43
556	PDE4A	Phosphodiesterase 4A	Protein Coding	45	GC19P010416	1.43
557	P2RY11	Purinergic Receptor P2Y11	Protein Coding	43	GC19P010146	1.43
558	CDC37	Cell Division Cycle 37	Protein Coding	41	GC19M010391	1.43
559	SLURP1	Secreted LY6/PLAUR Domain Containing 1	Protein Coding	41	GC08M142740	1.43
560	ICAM3	Intercellular Adhesion Molecule 3	Protein Coding	41	GC19M010335	1.43
561	ERC2	ELKS/RAB6-Interacting/CAST Family Member 2	Protein Coding	38	GC03M055509	1.43
562	JRK	Jrk Helix-Turn-Helix Protein	Protein Coding	36	GC08M142657	1.43
563	ATG4D	Autophagy Related 4D Cysteine Peptidase	Protein Coding	36	GC19P010543	1.43
564	LY6K	Lymphocyte Antigen 6 Family Member K	Protein Coding	34	GC08P142700	1.43
565	CCDC66	Coiled-Coil Domain Containing 66	Protein Coding	32	GC03P056567	1.43
566	THEM6	Thioesterase Superfamily Member 6	Protein Coding	31	GC08P142727	1.43
567	C19orf38	Chromosome 19 Open Reading Frame 38	Protein Coding	28	GC19P010837	1.43
568	FDX2	Ferredoxin 2	Protein Coding	28	GC19M010311	1.43
569	ENSG00000252045		RNA Gene	6	GC21P032538	1.43
570	RNA5SP490	RNA, 5S Ribosomal Pseudogene 490	Pseudogene	6	GC21P032563	1.43
571	lnc-JRK-1		RNA Gene	4	GC08M142685	1.43
572	lnc-TIMM29-1		RNA Gene	4	GC19P010385	1.43
573	ENSG00000285717		RNA Gene	4	GC06P006450	1.43
574	lnc-PDE4A-1		RNA Gene	3	GC19P010412	1.43
575	piR-52079-093		RNA Gene	3	GC19P010407	1.43
576	PLAU	Plasminogen Activator, Urokinase	Protein Coding	52	GC10P073909	1.42
577	NOX1	NADPH Oxidase 1	Protein Coding	42	GC0XM100843	1.39
578	MMP3	Matrix Metallopeptidase 3	Protein Coding	51	GC11M102835	1.31
579	MIR135B	MicroRNA 135b	RNA Gene	17	GC01M205448	1.31
580	S100A9	S100 Calcium Binding Protein A9	Protein Coding	43	GC01P153357	1.28
581	CLDN2	Claudin 2	Protein Coding	41	GC0XP106900	1.28
582	GKN1	Gastrokine 1	Protein Coding	38	GC02P068974	1.28
583	GJB1	Gap Junction Protein Beta 1	Protein Coding	47	GC0XP071215	1.27
584	IL18	Interleukin 18	Protein Coding	44	GC11M112143	1.27
585	IL22	Interleukin 22	Protein Coding	41	GC12M068248	1.27
586	CASP1	Caspase 1	Protein Coding	50	GC11M105025	1.21
587	BAX	BCL2 Associated X, Apoptosis Regulator	Protein Coding	48	GC19P048954	1.21
588	TREM1	Triggering Receptor Expressed On Myeloid Cells 1	Protein Coding	42	GC06M041267	1.17
589	GJA1	Gap Junction Protein Alpha 1	Protein Coding	50	GC06P121436	1.13
590	SERPINE1	Serpin Family E Member 1	Protein Coding	50	GC07P101127	1.13
591	PLAT	Plasminogen Activator, Tissue Type	Protein Coding	49	GC08M042174	1.13
592	IL12B	Interleukin 12B	Protein Coding	44	GC05M159314	1.13
593	CDX1	Caudal Type Homeobox 1	Protein Coding	36	GC05P150133	1.13
594	CCND1	Cyclin D1	Protein Coding	52	GC11P069641	1.13
595	HSPA2	Heat Shock Protein Family A (Hsp70) Member 2	Protein Coding	44	GC14P064535	1.09
596	CRH	Corticotropin Releasing Hormone	Protein Coding	43	GC08M066176	1.09
597	CLDN4	Claudin 4	Protein Coding	41	GC07P073799	1.09
598	CLDN11	Claudin 11	Protein Coding	40	GC03P170418	1.09
599	CLDN23	Claudin 23	Protein Coding	36	GC08P008701	1.09
600	CCL3	C-C Motif Chemokine Ligand 3	Protein Coding	39	GC17M036088	1.06
601	B2M	Beta-2-Microglobulin	Protein Coding	49	GC15P044711	1.04
602	POU5F1	POU Class 5 Homeobox 1	Protein Coding	47	GC06M031177	1.04
603	SELL	Selectin L	Protein Coding	42	GC01M169690	1.04
604	PTEN	Phosphatase And Tensin Homolog	Protein Coding	53	GC10P087863	1.03
605	CDKN1A	Cyclin Dependent Kinase Inhibitor 1A	Protein Coding	50	GC06P046057	1.03
606	CD209	CD209 Molecule	Protein Coding	42	GC19M007739	1.01
607	CD83	CD83 Molecule	Protein Coding	38	GC06P014117	1.01
608	TSLP	Thymic Stromal Lymphopoietin	Protein Coding	38	GC05P111070	0.99
609	TERC	Telomerase RNA Component	RNA Gene	27	GC03M169765	0.99
610	TFRC	Transferrin Receptor	Protein Coding	49	GC03M196027	0.98
611	FTL	Ferritin Light Chain	Protein Coding	47	GC19P048965	0.98
612	FPR2	Formyl Peptide Receptor 2	Protein Coding	46	GC19P051752	0.98
613	IL7	Interleukin 7	Protein Coding	42	GC08M078689	0.98

614	CHIA	Chitinase Acidic	Protein Coding	40	GC01P111291	0.98
615	FPR3	Formyl Peptide Receptor 3	Protein Coding	39	GC19P051795	0.98
616	MAPK1	Mitogen-Activated Protein Kinase 1	Protein Coding	51	GC22M021754	0.95
617	SLC5A5	Solute Carrier Family 5 Member 5	Protein Coding	45	GC19P022240	0.95
618	MKI67	Marker Of Proliferation Ki-67	Protein Coding	42	GC10M128096	0.95
619	TDGF1	Teratocarcinoma-Derived Growth Factor 1	Protein Coding	42	GC03P046576	0.95
620	MMP1	Matrix Metallopeptidase 1	Protein Coding	52	GC11M102810	0.92
621	SMAD4	SMAD Family Member 4	Protein Coding	50	GC18P051028	0.92
622	CYP3A4	Cytochrome P450 Family 3 Subfamily A Member 4	Protein Coding	49	GC07M099759	0.92
623	CD74	CD74 Molecule	Protein Coding	43	GC05M150378	0.92
624	AKT1	AKT Serine/Threonine Kinase 1	Protein Coding	54	GC14M104769	0.9
625	F2RL1	F2R Like Trypsin Receptor 1	Protein Coding	46	GC05P076818	0.9
626	CDC73	Cell Division Cycle 73	Protein Coding	45	GC01P193090	0.9
627	HSPA1B	Heat Shock Protein Family A (Hsp70) Member 1B	Protein Coding	41	GC06P033427	0.9
628	SMOX	Spermine Oxidase	Protein Coding	39	GC20P004120	0.9
629	HSP90AA2P	Heat Shock Protein 90 Alpha Family Class A Member 2, Pseudogene	Pseudogene	21	GC11M027888	0.9
630	EZH2	Enhancer Of Zeste 2 Polycomb Repressive Complex 2 Subunit	Protein Coding	53	GC07M148807	0.86
631	CTSB	Cathepsin B	Protein Coding	51	GC08M011842	0.86
632	CCNB1	Cyclin B1	Protein Coding	47	GC05P069167	0.86
633	CDK1	Cyclin Dependent Kinase 1	Protein Coding	46	GC10P060772	0.86
634	VCAM1	Vascular Cell Adhesion Molecule 1	Protein Coding	46	GC01P100719	0.86
635	SELP	Selectin P	Protein Coding	46	GC01M169558	0.86
636	CTSL	Cathepsin L	Protein Coding	46	GC09P087725	0.86
637	LIF	LIF Interleukin 6 Family Cytokine	Protein Coding	43	GC22M030240	0.86
638	S100A12	S100 Calcium Binding Protein A12	Protein Coding	39	GC01M153346	0.86
639	IRX1	Iroquois Homeobox 1	Protein Coding	36	GC05P003596	0.86
640	GATA4	GATA Binding Protein 4	Protein Coding	48	GC08P011676	0.83
641	GATA6	GATA Binding Protein 6	Protein Coding	47	GC18P022169	0.83
642	GCG	Glucagon	Protein Coding	41	GC02M162142	0.83
643	CXCR4	C-X-C Motif Chemokine Receptor 4	Protein Coding	52	GC02M136114	0.8
644	MAPK14	Mitogen-Activated Protein Kinase 14	Protein Coding	52	GC06P046047	0.8
645	COMT	Catechol-O-Methyltransferase	Protein Coding	50	GC22P019941	0.8
646	NOS3	Nitric Oxide Synthase 3	Protein Coding	50	GC07P150990	0.8
647	FOS	Fos Proto-Oncogene, AP-1 Transcription Factor Subunit	Protein Coding	50	GC14P075278	0.8
648	PBX1	PBX Homeobox 1	Protein Coding	49	GC01P164524	0.8
649	GLI2	GLI Family Zinc Finger 2	Protein Coding	48	GC02P120735	0.8
650	SMO	Smoothened, Frizzled Class Receptor	Protein Coding	48	GC07P129303	0.8
651	HPGD	15-Hydroxyprostaglandin Dehydrogenase	Protein Coding	48	GC04M174490	0.8
652	IL4R	Interleukin 4 Receptor	Protein Coding	47	GC16P027325	0.8
653	USP7	Ubiquitin Specific Peptidase 7	Protein Coding	47	GC16M008892	0.8
654	TNC	Tenascin C	Protein Coding	47	GC09M115019	0.8
655	CYBA	Cytochrome B-245 Alpha Chain	Protein Coding	46	GC16M088643	0.8
656	TYMP	Thymidine Phosphorylase	Protein Coding	46	GC22M050525	0.8
657	CD79A	CD79a Molecule	Protein Coding	46	GC19P041877	0.8
658	CARD11	Caspase Recruitment Domain Family Member 11	Protein Coding	46	GC07M002912	0.8
659	OGT	O-Linked N-Acetylglucosamine (GlcNAc) Transferase	Protein Coding	45	GC0XP071534	0.8
660	BCL10	BCL10 Immune Signaling Adaptor	Protein Coding	45	GC01M085265	0.8
661	EZR	Ezrin	Protein Coding	45	GC06M158765	0.8
662	TIMP2	TIMP Metallopeptidase Inhibitor 2	Protein Coding	44	GC17M078852	0.8
663	S100A4	S100 Calcium Binding Protein A4	Protein Coding	44	GC01M153543	0.8
664	BTC	Betacellulin	Protein Coding	44	GC04M074744	0.8
665	HAVCR2	Hepatitis A Virus Cellular Receptor 2	Protein Coding	43	GC05M157063	0.8
666	BMI1	BMI1 Proto-Oncogene, Polycomb Ring Finger	Protein Coding	43	GC10P022326	0.8
667	BAG1	BAG Cochaperone 1	Protein Coding	43	GC09M033245	0.8
668	CEACAM1	CEA Cell Adhesion Molecule 1	Protein Coding	42	GC19M042507	0.8
669	SLC7A2	Solute Carrier Family 7 Member 2	Protein Coding	42	GC08P017497	0.8
670	ACTR2	Actin Related Protein 2	Protein Coding	42	GC02P065227	0.8
671	ACTR3	Actin Related Protein 3	Protein Coding	42	GC02P113889	0.8
672	S100A2	S100 Calcium Binding Protein A2	Protein Coding	41	GC01M153561	0.8
673	MAGI1	Membrane Associated Guanylate Kinase, WW And PDZ Domain Containing 1	Protein Coding	41	GC03M065330	0.8
674	VTCN1	V-Set Domain Containing T Cell Activation Inhibitor 1	Protein Coding	40	GC01M117143	0.8
675	CD276	CD276 Molecule	Protein Coding	40	GC15P073683	0.8
676	UCN	Urocortin	Protein Coding	38	GC02M027308	0.8
677	MAGI3	Membrane Associated Guanylate Kinase, WW And PDZ Domain Containing 3	Protein Coding	35	GC01P113390	0.8
678	MIR24-2	MicroRNA 24-2	RNA Gene	16	GC19M013986	0.8
679	CYBB	Cytochrome B-245 Beta Chain	Protein Coding	47	GC0XP037780	0.77
680	AKR1A1	Aldo-Keto Reductase Family 1 Member A1	Protein Coding	43	GC01P045550	0.77
681	HDAC6	Histone Deacetylase 6	Protein Coding	52	GC0XP048801	0.74
682	SRC	SRC Proto-Oncogene, Non-Receptor Tyrosine Kinase	Protein Coding	52	GC20P037344	0.74
683	PLA2G4A	Phospholipase A2 Group IVA	Protein Coding	49	GC01P186798	0.74
684	CHRM3	Cholinergic Receptor Muscarinic 3	Protein Coding	48	GC01P239386	0.74
685	HSP90AA1	Heat Shock Protein 90 Alpha Family Class A Member 1	Protein Coding	48	GC14M102080	0.74
686	DAPK1	Death Associated Protein Kinase 1	Protein Coding	48	GC09P087497	0.74
687	GLI1	GLI Family Zinc Finger 1	Protein Coding	47	GC12P057460	0.74
688	TP63	Tumor Protein P63	Protein Coding	47	GC03P189566	0.74
689	IFIH1	Interferon Induced With Helicase C Domain 1	Protein Coding	47	GC02M162267	0.74
690	FPR1	Formyl Peptide Receptor 1	Protein Coding	47	GC19M051745	0.74

691	PLA2G1B	Phospholipase A2 Group IB	Protein Coding	46	GC12M120322	0.74
692	CASP4	Caspase 4	Protein Coding	46	GC11M104942	0.74
693	BIRC3	Baculoviral IAP Repeat Containing 3	Protein Coding	46	GC11P102317	0.74
694	ENG	Endoglin	Protein Coding	46	GC09M127815	0.74
695	POU2F1	POU Class 2 Homeobox 1	Protein Coding	45	GC01P167190	0.74
696	PIAS1	Protein Inhibitor Of Activated STAT 1	Protein Coding	45	GC15P068054	0.74
697	BRD4	Bromodomain Containing 4	Protein Coding	45	GC19M015236	0.74
698	SFTPD	Surfactant Protein D	Protein Coding	44	GC10M079937	0.74
699	HSD17B2	Hydroxysteroid 17-Beta Dehydrogenase 2	Protein Coding	44	GC16P082068	0.74
700	CD34	CD34 Molecule	Protein Coding	44	GC01M207880	0.74
701	CXCR1	C-X-C Motif Chemokine Receptor 1	Protein Coding	43	GC02M218162	0.74
702	HSPA1L	Heat Shock Protein Family A (Hsp70) Member 1 Like	Protein Coding	43	GC06M031809	0.74
703	PPP1R1B	Protein Phosphatase 1 Regulatory Inhibitor Subunit 1B	Protein Coding	43	GC17P039626	0.74
704	THBS4	Thrombospondin 4	Protein Coding	42	GC05P079991	0.74
705	LIPF	Lipase F, Gastric Type	Protein Coding	42	GC10P088664	0.74
706	CD63	CD63 Molecule	Protein Coding	42	GC12M055725	0.74
707	FGF7	Fibroblast Growth Factor 7	Protein Coding	42	GC15P049423	0.74
708	CXCR5	C-X-C Motif Chemokine Receptor 5	Protein Coding	41	GC11P118892	0.74
709	CXCL13	C-X-C Motif Chemokine Ligand 13	Protein Coding	41	GC04P077511	0.74
710	HCRT	Hypocretin Neuropeptide Precursor	Protein Coding	41	GC17M042185	0.74
711	DROSHA	Drosha Ribonuclease III	Protein Coding	41	GC05M031401	0.74
712	REG4	Regenerating Family Member 4	Protein Coding	40	GC01M119794	0.74
713	CCNC	Cyclin C	Protein Coding	40	GC06M099542	0.74
714	HOXB7	Homeobox B7	Protein Coding	40	GC17M048607	0.74
715	FOXD3	Forkhead Box D3	Protein Coding	39	GC01P063323	0.74
716	MAGEA1	MAGE Family Member A1	Protein Coding	38	GC0XP153179	0.74
717	IRGM	Immunity Related GTPase M	Protein Coding	37	GC05P150846	0.74
718	OMP	Olfactory Marker Protein	Protein Coding	37	GC11P077102	0.74
719	ABHD11	Abhydrolase Domain Containing 11	Protein Coding	37	GC07M073736	0.74
720	GPR162	G Protein-Coupled Receptor 162	Protein Coding	36	GC12P006821	0.74
721	CDR1	Cerebellar Degeneration Related Protein 1	Protein Coding	31	GC0XM140782	0.74
722	CD24	CD24 Molecule	Protein Coding	30	GC06M106969	0.74
723	NKX6-3	NK6 Homeobox 3	Protein Coding	28	GC08M041645	0.74
724	MIR133A1	MicroRNA 133a-1	RNA Gene	15	GC18M021826	0.74
725	ABHD11-AS1	ABHD11 Antisense RNA 1 (Tail To Tail)	RNA Gene	15	GC07P073735	0.74
726	HDAC2	Histone Deacetylase 2	Protein Coding	52	GC06M113933	0.65
727	ARG1	Arginase 1	Protein Coding	50	GC06P131473	0.65
728	FN1	Fibronectin 1	Protein Coding	50	GC02M215360	0.65
729	AURKA	Aurora Kinase A	Protein Coding	50	GC20M056370	0.65
730	STK11	Serine/Threonine Kinase 11	Protein Coding	49	GC19P001177	0.65
731	PRKCZ	Protein Kinase C Zeta	Protein Coding	48	GC01P002050	0.65
732	CD40	CD40 Molecule	Protein Coding	48	GC20P046118	0.65
733	TCF7L2	Transcription Factor 7 Like 2	Protein Coding	47	GC10P112950	0.65
734	CD3E	CD3e Molecule	Protein Coding	47	GC11P118304	0.65
735	EDN1	Endothelin 1	Protein Coding	47	GC06P012290	0.65
736	EEF2K	Eukaryotic Elongation Factor 2 Kinase	Protein Coding	47	GC16P022217	0.65
737	PRDX2	Peroxiredoxin 2	Protein Coding	46	GC19M012796	0.65
738	STAT4	Signal Transducer And Activator Of Transcription 4	Protein Coding	46	GC02M191029	0.65
739	ARG2	Arginase 2	Protein Coding	46	GC14P067619	0.65
740	IL12RB1	Interleukin 12 Receptor Subunit Beta 1	Protein Coding	45	GC19M018030	0.65
741	CSF2	Colony Stimulating Factor 2	Protein Coding	44	GC05P132073	0.65
742	MMP24	Matrix Metallopeptidase 24	Protein Coding	44	GC20P035226	0.65
743	FOXM1	Forkhead Box M1	Protein Coding	44	GC12M002857	0.65
744	HP	Haptoglobin	Protein Coding	44	GC16P072089	0.65
745	HOXA1	Homeobox A1	Protein Coding	43	GC07M027092	0.65
746	MMP12	Matrix Metallopeptidase 12	Protein Coding	43	GC11M102862	0.65
747	GJA4	Gap Junction Protein Alpha 4	Protein Coding	43	GC01P034792	0.65
748	CHIT1	Chitinase 1	Protein Coding	43	GC01M203181	0.65
749	IL12RB2	Interleukin 12 Receptor Subunit Beta 2	Protein Coding	43	GC01P067307	0.65
750	KLF5	Kruppel Like Factor 5	Protein Coding	43	GC13P073054	0.65
751	TNFRSF8	TNF Receptor Superfamily Member 8	Protein Coding	43	GC01P012063	0.65
752	IL9	Interleukin 9	Protein Coding	43	GC05M135891	0.65
753	HOXA10	Homeobox A10	Protein Coding	42	GC07M027229	0.65
754	CEACAM5	CEA Cell Adhesion Molecule 5	Protein Coding	42	GC19P041709	0.65
755	TERF1	Telomeric Repeat Binding Factor 1	Protein Coding	42	GC08P073003	0.65
756	NR0B2	Nuclear Receptor Subfamily 0 Group B Member 2	Protein Coding	42	GC01M026922	0.65
757	SATB1	SATB Homeobox 1	Protein Coding	42	GC03M018364	0.65
758	IGF2BP3	Insulin Like Growth Factor 2 MRNA Binding Protein 3	Protein Coding	42	GC07M023316	0.65
759	CLDN5	Claudin 5	Protein Coding	41	GC22M019523	0.65
760	HSPE1	Heat Shock Protein Family E (Hsp10) Member 1	Protein Coding	40	GC02P197501	0.65
761	MMP25	Matrix Metallopeptidase 25	Protein Coding	39	GC16P004057	0.65
762	LLGL2	LLGL Scribble Cell Polarity Complex Component 2	Protein Coding	39	GC17P075525	0.65
763	GATA5	GATA Binding Protein 5	Protein Coding	39	GC20M062464	0.65
764	LAMP3	Lysosomal Associated Membrane Protein 3	Protein Coding	38	GC03M183122	0.65
765	NOXA1	NADPH Oxidase Activator 1	Protein Coding	38	GC09P137423	0.65
766	NOXO1	NADPH Oxidase Organizer 1	Protein Coding	38	GC16M002164	0.65
767	TRIM31	Tripartite Motif Containing 31	Protein Coding	37	GC06M030652	0.65

768	NTAN1	N-Terminal Asparagine Amidase	Protein Coding	36	GC16M015037	0.65
769	TIFA	TRAF Interacting Protein With Forkhead Associated Domain	Protein Coding	35	GC04M112274	0.65
770	RIOX2	Ribosomal Oxygenase 2	Protein Coding	31	GC03M097942	0.65
771	DEFB103B	Defensin Beta 103B	Protein Coding	30	GC08M007430	0.65
772	MIR370	MicroRNA 370	RNA Gene	19	GC14P104253	0.65
773	TF	Transferrin	Protein Coding	49	GC03P133666	0.63
774	DUOX2	Dual Oxidase 2	Protein Coding	43	GC15M045092	0.63
775	MOS	MOS Proto-Oncogene, Serine/Threonine Kinase	Protein Coding	39	GC08M056112	0.63
776	LPO	Lactoperoxidase	Protein Coding	38	GC17P058218	0.63
777	DMBT1	Deleted In Malignant Brain Tumors 1	Protein Coding	41	GC10P122560	0.46
778	FUBP1	Far Upstream Element Binding Protein 1	Protein Coding	39	GC01M077944	0.46
779	IFI27	Interferon Alpha Inducible Protein 27	Protein Coding	38	GC14P094104	0.46
780	MIRLET7C	MicroRNA Let-7c	RNA Gene	19	GC21P016553	0.46
781	LOC1113651	NOS2 5' Regulatory Region	Biological Region	2	GC17U902748	0.46
782	MMP14	Matrix Metallopeptidase 14	Protein Coding	51	GC14P025005	0.44
783	DNMT1	DNA Methyltransferase 1	Protein Coding	51	GC19M010133	0.44
784	DNMT3A	DNA Methyltransferase 3 Alpha	Protein Coding	51	GC02M025228	0.44
785	CHEK1	Checkpoint Kinase 1	Protein Coding	50	GC11P125625	0.44
786	PRKCH	Protein Kinase C Eta	Protein Coding	50	GC14P061187	0.44
787	SYK	Spleen Associated Tyrosine Kinase	Protein Coding	50	GC09P091113	0.44
788	SNCA	Synuclein Alpha	Protein Coding	50	GC04M089724	0.44
789	G6PD	Glucose-6-Phosphate Dehydrogenase	Protein Coding	50	GC0XM154531	0.44
790	TTR	Transthyretin	Protein Coding	49	GC18P031557	0.44
791	PLK1	Polo Like Kinase 1	Protein Coding	49	GC16P023869	0.44
792	NOTCH3	Notch Receptor 3	Protein Coding	49	GC19M015159	0.44
793	DNMT3B	DNA Methyltransferase 3 Beta	Protein Coding	49	GC20P032762	0.44
794	ZEB1	Zinc Finger E-Box Binding Homeobox 1	Protein Coding	48	GC10P031318	0.44
795	SLC6A4	Solute Carrier Family 6 Member 4	Protein Coding	48	GC17M030194	0.44
796	CYP2A6	Cytochrome P450 Family 2 Subfamily A Member 6	Protein Coding	47	GC19M040843	0.44
797	TKT	Transketolase	Protein Coding	47	GC03M053224	0.44
798	TCF4	Transcription Factor 4	Protein Coding	46	GC18M055222	0.44
799	TNFRSF10A	TNF Receptor Superfamily Member 10a	Protein Coding	45	GC08M023190	0.44
800	LAMA1	Laminin Subunit Alpha 1	Protein Coding	45	GC18M006941	0.44
801	F3	Coagulation Factor III, Tissue Factor	Protein Coding	45	GC01M094530	0.44
802	RORC	RAR Related Orphan Receptor C	Protein Coding	45	GC01M151806	0.44
803	BDKRB2	Bradykinin Receptor B2	Protein Coding	45	GC14P096205	0.44
804	HES1	Hes Family BHLH Transcription Factor 1	Protein Coding	44	GC03P194136	0.44
805	GLP2R	Glucagon Like Peptide 2 Receptor	Protein Coding	44	GC17P009822	0.44
806	TNFSF13	TNF Superfamily Member 13	Protein Coding	44	GC17P007558	0.44
807	SERPINB2	Serpin Family B Member 2	Protein Coding	44	GC18P063871	0.44
808	SOCS3	Suppressor Of Cytokine Signaling 3	Protein Coding	44	GC17M078356	0.44
809	SOCS2	Suppressor Of Cytokine Signaling 2	Protein Coding	43	GC12P093569	0.44
810	BDKRB1	Bradykinin Receptor B1	Protein Coding	43	GC14P096260	0.44
811	HMMR	Hyaluronan Mediated Motility Receptor	Protein Coding	42	GC05P163480	0.44
812	IL21R	Interleukin 21 Receptor	Protein Coding	42	GC16P027413	0.44
813	PPIG	Peptidylprolyl Isomerase G	Protein Coding	42	GC02P169584	0.44
814	ARNTL	Aryl Hydrocarbon Receptor Nuclear Translocator Like	Protein Coding	42	GC11P013276	0.44
815	LIN28A	Lin-28 Homolog A	Protein Coding	41	GC01P026410	0.44
816	NMB	Neuromedin B	Protein Coding	41	GC15M084655	0.44
817	AAAS	Aladin WD Repeat Nucleoporin	Protein Coding	41	GC12M053307	0.44
818	LAPTM4B	Lysosomal Protein Transmembrane 4 Beta	Protein Coding	39	GC08P097775	0.44
819	TXNDC5	Thioredoxin Domain Containing 5	Protein Coding	39	GC06M007893	0.44
820	ERGIC1	Endoplasmic Reticulum-Golgi Intermediate Compartment 1	Protein Coding	39	GC05P172834	0.44
821	CEACAM7	CEA Cell Adhesion Molecule 7	Protein Coding	37	GC19M041673	0.44
822	APLN	Apelin	Protein Coding	37	GC0XM129645	0.44
823	ADGRB1	Adhesion G Protein-Coupled Receptor B1	Protein Coding	34	GC08P142449	0.44
824	MPIG6B	Megakaryocyte And Platelet Inhibitory Receptor G6b	Protein Coding	31	GC06P033627	0.44
825	MIR503	MicroRNA 503	RNA Gene	15	GC0XM134624	0.44
826	MIR421	MicroRNA 421	RNA Gene	12	GC0XM074218	0.44
827	XS	X-Linked Suppressor Of LU Antigens	Genetic Locus	5	GC0XU990239	0.44
828	LOC1108062	Solute Carrier Family 6 Member 4 Gene Promoter	Biological Region	2	GC17U902739	0.44
829	HNF4A	Hepatocyte Nuclear Factor 4 Alpha	Protein Coding	50	GC20P044355	0.41
830	RB1	RB Transcriptional Corepressor 1	Protein Coding	49	GC13P048303	0.41
831	CAV1	Caveolin 1	Protein Coding	48	GC07P116524	0.41
832	SYP	Synaptophysin	Protein Coding	43	GC0XM049187	0.41
833	SMAD7	SMAD Family Member 7	Protein Coding	43	GC18M048919	0.41
834	MAP2K1	Mitogen-Activated Protein Kinase Kinase 1	Protein Coding	54	GC15P066386	0.36
835	ABL1	ABL Proto-Oncogene 1, Non-Receptor Tyrosine Kinase	Protein Coding	53	GC09P130713	0.36
836	TGFBR2	Transforming Growth Factor Beta Receptor 2	Protein Coding	52	GC03P030623	0.36
837	NOTCH1	Notch Receptor 1	Protein Coding	51	GC09M136582	0.36
838	CCND2	Cyclin D2	Protein Coding	50	GC12P006576	0.36
839	IGF1	Insulin Like Growth Factor 1	Protein Coding	49	GC12M102395	0.36
840	NOTCH2	Notch Receptor 2	Protein Coding	49	GC01M119911	0.36
841	SMAD3	SMAD Family Member 3	Protein Coding	48	GC15P067063	0.36
842	HIF1A	Hypoxia Inducible Factor 1 Subunit Alpha	Protein Coding	47	GC14P061695	0.36
843	CDKN2B	Cyclin Dependent Kinase Inhibitor 2B	Protein Coding	47	GC09M022002	0.36
844	IGF2	Insulin Like Growth Factor 2	Protein Coding	47	GC11M002130	0.36

845	YAP1	Yes Associated Protein 1	Protein Coding	47	GC11P102110	0.36
846	ENO2	Enolase 2	Protein Coding	47	GC12P006913	0.36
847	FGF2	Fibroblast Growth Factor 2	Protein Coding	47	GC04P122826	0.36
848	COL4A3	Collagen Type IV Alpha 3 Chain	Protein Coding	45	GC02P227164	0.36
849	CD274	CD274 Molecule	Protein Coding	45	GC09P005450	0.36
850	LCN2	Lipocalin 2	Protein Coding	44	GC09P128149	0.36
851	MBD3	Methyl-CpG Binding Domain Protein 3	Protein Coding	42	GC19M001690	0.36
852	IL17F	Interleukin 17F	Protein Coding	41	GC06M052209	0.36
853	MBD2	Methyl-CpG Binding Domain Protein 2	Protein Coding	41	GC18M054151	0.36
854	BIRC7	Baculoviral IAP Repeat Containing 7	Protein Coding	40	GC20P063235	0.36
855	PCDH10	Protocadherin 10	Protein Coding	39	GC04P133149	0.36
856	MUC7	Mucin 7, Secreted	Protein Coding	38	GC04P070430	0.36
857	A4GNT	Alpha-1,4-N-Acetylglucosaminyltransferase	Protein Coding	35	GC03M138123	0.36
858	MIR137	MicroRNA 137	RNA Gene	16	GC01M098046	0.36
859	ITGB2	Integrin Subunit Beta 2	Protein Coding	50	GC21M044885	0.29
860	VIM	Vimentin	Protein Coding	50	GC10P017227	0.29
861	MYD88	MYD88 Innate Immune Signal Transduction Adaptor	Protein Coding	50	GC03P038179	0.29
862	PTK2B	Protein Tyrosine Kinase 2 Beta	Protein Coding	49	GC08P027311	0.29
863	GATA3	GATA Binding Protein 3	Protein Coding	49	GC10P008045	0.29
864	CD247	CD247 Molecule	Protein Coding	49	GC01M167399	0.29
865	AHR	Aryl Hydrocarbon Receptor	Protein Coding	49	GC07P016916	0.29
866	CBS	Cystathionine Beta-Synthase	Protein Coding	48	GC21M043053	0.29
867	GRN	Granulin Precursor	Protein Coding	47	GC17P044345	0.29
868	TRAF6	TNF Receptor Associated Factor 6	Protein Coding	47	GC11M036467	0.29
869	PTK2	Protein Tyrosine Kinase 2	Protein Coding	47	GC08M140657	0.29
870	GPX1	Glutathione Peroxidase 1	Protein Coding	47	GC03M049370	0.29
871	CASP9	Caspase 9	Protein Coding	47	GC01M015491	0.29
872	MCL1	MCL1 Apoptosis Regulator, BCL2 Family Member	Protein Coding	47	GC01M150673	0.29
873	KLK3	Kallikrein Related Peptidase 3	Protein Coding	46	GC19P050854	0.29
874	VHL	Von Hippel-Lindau Tumor Suppressor	Protein Coding	46	GC03P010205	0.29
875	F2R	Coagulation Factor II Thrombin Receptor	Protein Coding	46	GC05P076716	0.29
876	NPPA	Natriuretic Peptide A	Protein Coding	46	GC01M011846	0.29
877	BAD	BCL2 Associated Agonist Of Cell Death	Protein Coding	46	GC11M064273	0.29
878	PIK3C2A	Phosphatidylinositol-4-Phosphate 3-Kinase Catalytic Subunit Type 2 Alpha	Protein Coding	45	GC11M017165	0.29
879	IDO1	Indoleamine 2,3-Dioxygenase 1	Protein Coding	45	GC08P039891	0.29
880	F5	Coagulation Factor V	Protein Coding	45	GC01M169511	0.29
881	WNT2	Wnt Family Member 2	Protein Coding	44	GC07M117276	0.29
882	CD9	CD9 Molecule	Protein Coding	44	GC12P006594	0.29
883	ITGAX	Integrin Subunit Alpha X	Protein Coding	43	GC16P031432	0.29
884	IRF4	Interferon Regulatory Factor 4	Protein Coding	43	GC06P000391	0.29
885	PYY	Peptide YY	Protein Coding	43	GC17M043952	0.29
886	LGR5	Leucine Rich Repeat Containing G Protein-Coupled Receptor 5	Protein Coding	43	GC12P071439	0.29
887	PAM	Peptidylglycine Alpha-Amidating Monooxygenase	Protein Coding	43	GC05P102753	0.29
888	MAP3K14	Mitogen-Activated Protein Kinase Kinase Kinase 14	Protein Coding	43	GC17M045263	0.29
889	BMP6	Bone Morphogenetic Protein 6	Protein Coding	43	GC06P007726	0.29
890	MARK2	Microtubule Affinity Regulating Kinase 2	Protein Coding	43	GC11P063838	0.29
891	LTBR	Lymphotoxin Beta Receptor	Protein Coding	42	GC12P006375	0.29
892	CHGB	Chromogranin B	Protein Coding	41	GC20P005911	0.29
893	IL1RL1	Interleukin 1 Receptor Like 1	Protein Coding	41	GC02P102294	0.29
894	TUSC3	Tumor Suppressor Candidate 3	Protein Coding	41	GC08P015417	0.29
895	REG1A	Regenerating Family Member 1 Alpha	Protein Coding	41	GC02P079120	0.29
896	GPT	Glutamic--Pyruvic Transaminase	Protein Coding	41	GC08P144502	0.29
897	CCL4	C-C Motif Chemokine Ligand 4	Protein Coding	41	GC17P036103	0.29
898	BCL9	BCL9 Transcription Coactivator	Protein Coding	41	GC01P147541	0.29
899	UBD	Ubiquitin D	Protein Coding	39	GC06M029556	0.29
900	NTS	Neurotensin	Protein Coding	39	GC12P085876	0.29
901	PCDH17	Protocadherin 17	Protein Coding	36	GC13P057630	0.29
902	DEFA6	Defensin Alpha 6	Protein Coding	36	GC08M006924	0.29
903	MLN	Motilin	Protein Coding	35	GC06M033794	0.29
904	ZNF318	Zinc Finger Protein 318	Protein Coding	35	GC06M043309	0.29
905	TNFAIP8L2	TNF Alpha Induced Protein 8 Like 2	Protein Coding	34	GC01P151129	0.29
906	RPRM	Reprimo, TP53 Dependent G2 Arrest Mediator Homolog	Protein Coding	33	GC02M153477	0.29
907	SLFN5	Schlafen Family Member 5	Protein Coding	32	GC17P035243	0.29
908	MIR499A	MicroRNA 499a	RNA Gene	20	GC20P034990	0.29
909	MIR34B	MicroRNA 34b	RNA Gene	19	GC11P111578	0.29
910	MIR210	MicroRNA 210	RNA Gene	19	GC11M000622	0.29
911	MIR222	MicroRNA 222	RNA Gene	19	GC0XM045747	0.29
912	MIR223	MicroRNA 223	RNA Gene	19	GC0XP066018	0.29
913	MIR196A2	MicroRNA 196a-2	RNA Gene	19	GC12P054167	0.29
914	MIR9-3	MicroRNA 9-3	RNA Gene	17	GC15P089363	0.29
915	MIR129-2	MicroRNA 129-2	RNA Gene	16	GC11P043633	0.29
916	TGFBR1	Transforming Growth Factor Beta Receptor 1	Protein Coding	53	GC09P099104	0.21
917	IKBKB	Inhibitor Of Nuclear Factor Kappa B Kinase Subunit Beta	Protein Coding	53	GC08P042271	0.21
918	MAP2K2	Mitogen-Activated Protein Kinase Kinase 2	Protein Coding	53	GC19M004090	0.21
919	AR	Androgen Receptor	Protein Coding	53	GC0XP067544	0.21
920	UCHL1	Ubiquitin C-Terminal Hydrolase L1	Protein Coding	52	GC04P041256	0.21
921	SLC2A1	Solute Carrier Family 2 Member 1	Protein Coding	52	GC01M042925	0.21

922	VDR	Vitamin D Receptor	Protein Coding	52	GC12M047841	0.21
923	PIK3CA	Phosphatidylinositol-4,5-Bisphosphate 3-Kinase Catalytic Subunit Alpha	Protein Coding	52	GC03P179148	0.21
924	CA2	Carbonic Anhydrase 2	Protein Coding	52	GC08P085463	0.21
925	CFTR	CF Transmembrane Conductance Regulator	Protein Coding	51	GC07P117465	0.21
926	RAD51	RAD51 Recombinase	Protein Coding	51	GC15P040694	0.21
927	MAP3K7	Mitogen-Activated Protein Kinase Kinase Kinase 7	Protein Coding	51	GC06M090513	0.21
928	ALK	ALK Receptor Tyrosine Kinase	Protein Coding	51	GC02M029156	0.21
929	CTH	Cystathionine Gamma-Lyase	Protein Coding	50	GC01P070411	0.21
930	GLUD1	Glutamate Dehydrogenase 1	Protein Coding	50	GC10M087050	0.21
931	ITGB1	Integrin Subunit Beta 1	Protein Coding	50	GC10M032900	0.21
932	TGFB2	Transforming Growth Factor Beta 2	Protein Coding	50	GC01P218345	0.21
933	GSK3B	Glycogen Synthase Kinase 3 Beta	Protein Coding	50	GC03M119821	0.21
934	PGR	Progesterone Receptor	Protein Coding	50	GC11M100943	0.21
935	MAPK8	Mitogen-Activated Protein Kinase 8	Protein Coding	50	GC10P048306	0.21
936	LPL	Lipoprotein Lipase	Protein Coding	50	GC08P019901	0.21
937	CYCS	Cytochrome C, Somatic	Protein Coding	49	GC07M025118	0.21
938	SQSTM1	Sequestosome 1	Protein Coding	49	GC05P179806	0.21
939	PRKDC	Protein Kinase, DNA-Activated, Catalytic Subunit	Protein Coding	49	GC08M047773	0.21
940	IRAK1	Interleukin 1 Receptor Associated Kinase 1	Protein Coding	49	GC0XM154010	0.21
941	NQO1	NAD(P)H Quinone Dehydrogenase 1	Protein Coding	49	GC16M069706	0.21
942	EPOR	Erythropoietin Receptor	Protein Coding	49	GC19M011377	0.21
943	MAPK3	Mitogen-Activated Protein Kinase 3	Protein Coding	49	GC16M030117	0.21
944	CASP6	Caspase 6	Protein Coding	49	GC04M109688	0.21
945	CAPN1	Calpain 1	Protein Coding	49	GC11P065198	0.21
946	CYP2C9	Cytochrome P450 Family 2 Subfamily C Member 9	Protein Coding	49	GC10P094938	0.21
947	ROCK1	Rho Associated Coiled-Coil Containing Protein Kinase 1	Protein Coding	49	GC18M020946	0.21
948	CTSK	Cathepsin K	Protein Coding	49	GC01M150796	0.21
949	AURKB	Aurora Kinase B	Protein Coding	49	GC17M008770	0.21
950	AGT	Angiotensinogen	Protein Coding	49	GC01M230702	0.21
951	CXCR2	C-X-C Motif Chemokine Receptor 2	Protein Coding	48	GC02P218125	0.21
952	ITGA5	Integrin Subunit Alpha 5	Protein Coding	48	GC12M054396	0.21
953	KAT2B	Lysine Acetyltransferase 2B	Protein Coding	48	GC03P020081	0.21
954	NF1	Neurofibromin 1	Protein Coding	48	GC17P031007	0.21
955	CDKN1C	Cyclin Dependent Kinase Inhibitor 1C	Protein Coding	47	GC11M002886	0.21
956	GPI	Glucose-6-Phosphate Isomerase	Protein Coding	47	GC19P034359	0.21
957	CSK	C-Terminal Src Kinase	Protein Coding	47	GC15P074782	0.21
958	KDM1A	Lysine Demethylase 1A	Protein Coding	47	GC01P023019	0.21
959	LDHB	Lactate Dehydrogenase B	Protein Coding	47	GC12M021635	0.21
960	PDCD1	Programmed Cell Death 1	Protein Coding	47	GC02M241849	0.21
961	RAB7A	RAB7A, Member RAS Oncogene Family	Protein Coding	47	GC03P128737	0.21
962	WNT3A	Wnt Family Member 3A	Protein Coding	47	GC01P228072	0.21
963	LMNB1	Lamin B1	Protein Coding	47	GC05P126776	0.21
964	SERPINC1	Serpin Family C Member 1	Protein Coding	47	GC01M174131	0.21
965	CLDN1	Claudin 1	Protein Coding	47	GC03M190305	0.21
966	PLCB4	Phospholipase C Beta 4	Protein Coding	47	GC20P009024	0.21
967	PLCG1	Phospholipase C Gamma 1	Protein Coding	47	GC20P041136	0.21
968	PNLIP	Pancreatic Lipase	Protein Coding	47	GC10P116545	0.21
969	GGT1	Gamma-Glutamyltransferase 1	Protein Coding	47	GC22P024583	0.21
970	MAP4K4	Mitogen-Activated Protein Kinase Kinase Kinase Kinase 4	Protein Coding	47	GC02P101773	0.21
971	CAMK2G	Calcium/Calmodulin Dependent Protein Kinase II Gamma	Protein Coding	47	GC10M073812	0.21
972	MAP3K5	Mitogen-Activated Protein Kinase Kinase Kinase 5	Protein Coding	47	GC06M136557	0.21
973	LRP6	LDL Receptor Related Protein 6	Protein Coding	47	GC12M013402	0.21
974	DES	Desmin	Protein Coding	47	GC02P219418	0.21
975	ENO1	Enolase 1	Protein Coding	47	GC01M008861	0.21
976	NLRP3	NLR Family Pyrin Domain Containing 3	Protein Coding	47	GC01P247415	0.21
977	EPCAM	Epithelial Cell Adhesion Molecule	Protein Coding	47	GC02P047345	0.21
978	CUL3	Cullin 3	Protein Coding	46	GC02M224470	0.21
979	CNR1	Cannabinoid Receptor 1	Protein Coding	46	GC06M088139	0.21
980	CYP1A1	Cytochrome P450 Family 1 Subfamily A Member 1	Protein Coding	46	GC15M074719	0.21
981	TGFB3	Transforming Growth Factor Beta 3	Protein Coding	46	GC14M075958	0.21
982	PTPN2	Protein Tyrosine Phosphatase Non-Receptor Type 2	Protein Coding	46	GC18M016781	0.21
983	TRAF3	TNF Receptor Associated Factor 3	Protein Coding	46	GC14P104312	0.21
984	IL1R1	Interleukin 1 Receptor Type 1	Protein Coding	46	GC02P102136	0.21
985	IL6ST	Interleukin 6 Signal Transducer	Protein Coding	46	GC05M055935	0.21
986	SLC18A2	Solute Carrier Family 18 Member A2	Protein Coding	46	GC10P117241	0.21
987	SLC19A1	Solute Carrier Family 19 Member 1	Protein Coding	46	GC21M045493	0.21
988	SH2B3	SH2B Adaptor Protein 3	Protein Coding	46	GC12P111405	0.21
989	SMAD2	SMAD Family Member 2	Protein Coding	46	GC18M047809	0.21
990	PHKA2	Phosphorylase Kinase Regulatory Subunit Alpha 2	Protein Coding	46	GC0XM018892	0.21
991	ANXA5	Annexin A5	Protein Coding	46	GC04M121667	0.21
992	FUCA1	Alpha-L-Fucosidase 1	Protein Coding	46	GC01M023845	0.21
993	DVL3	Dishevelled Segment Polarity Protein 3	Protein Coding	46	GC03P184155	0.21
994	DLL4	Delta Like Canonical Notch Ligand 4	Protein Coding	46	GC15P040929	0.21
995	MS4A1	Membrane Spanning 4-Domains A1	Protein Coding	46	GC11P060474	0.21
996	FCGR2A	Fc Fragment Of IgG Receptor IIa	Protein Coding	46	GC01P161505	0.21
997	BID	BH3 Interacting Domain Death Agonist	Protein Coding	46	GC22M017734	0.21
998	AFP	Alpha Fetoprotein	Protein Coding	46	GC04P073431	0.21

999	HMGCR	3-Hydroxy-3-Methylglutaryl-CoA Reductase	Protein Coding	45	GC05P075336	0.21
1000	CNR2	Cannabinoid Receptor 2	Protein Coding	45	GC01M023870	0.21
1001	CXCL12	C-X-C Motif Chemokine Ligand 12	Protein Coding	45	GC10M044370	0.21
1002	SUOX	Sulfite Oxidase	Protein Coding	45	GC12P055997	0.21
1003	PTPRB	Protein Tyrosine Phosphatase Receptor Type B	Protein Coding	45	GC12M070516	0.21
1004	SLC18A1	Solute Carrier Family 18 Member A1	Protein Coding	45	GC08M020144	0.21
1005	CIITA	Class II Major Histocompatibility Complex Transactivator	Protein Coding	45	GC16P010879	0.21
1006	PEPD	Peptidase D	Protein Coding	45	GC19M033386	0.21
1007	HSP90AB1	Heat Shock Protein 90 Alpha Family Class B Member 1	Protein Coding	45	GC06P044246	0.21
1008	F2RL3	F2R Like Thrombin Or Trypsin Receptor 3	Protein Coding	45	GC19P016888	0.21
1009	DVL2	Dishevelled Segment Polarity Protein 2	Protein Coding	45	GC17M007225	0.21
1010	MTHFD1	Methylenetetrahydrofolate Dehydrogenase, Cyclohydrolase And Formyltetrahydrofolate	Protein Coding	45	GC14P064388	0.21
1011	DDIT3	DNA Damage Inducible Transcript 3	Protein Coding	45	GC12M057516	0.21
1012	BCL6	BCL6 Transcription Repressor	Protein Coding	45	GC03M187721	0.21
1013	FHIT	Fragile Histidine Triad Diadenosine Triphosphatase	Protein Coding	45	GC03M059747	0.21
1014	HMGB1	High Mobility Group Box 1	Protein Coding	44	GC13M030456	0.21
1015	SLC17A5	Solute Carrier Family 17 Member 5	Protein Coding	44	GC06M073593	0.21
1016	SDC4	Syndecan 4	Protein Coding	44	GC20M045325	0.21
1017	SI	Sucrase-Isomaltase	Protein Coding	44	GC03M164978	0.21
1018	SP1	Sp1 Transcription Factor	Protein Coding	44	GC12P053380	0.21
1019	HSPA6	Heat Shock Protein Family A (Hsp70) Member 6	Protein Coding	44	GC01P161524	0.21
1020	IGFBP2	Insulin Like Growth Factor Binding Protein 2	Protein Coding	44	GC02P216632	0.21
1021	FGF4	Fibroblast Growth Factor 4	Protein Coding	44	GC11M069762	0.21
1022	AGER	Advanced Glycosylation End-Product Specific Receptor	Protein Coding	44	GC06M032180	0.21
1023	CPOX	Coproporphyrinogen Oxidase	Protein Coding	43	GC03M098576	0.21
1024	CEBPB	CCAAT Enhancer Binding Protein Beta	Protein Coding	43	GC20P050190	0.21
1025	PTPRN	Protein Tyrosine Phosphatase Receptor Type N	Protein Coding	43	GC02M219289	0.21
1026	TRIB3	Tribbles Pseudokinase 3	Protein Coding	43	GC20P000361	0.21
1027	TLR6	Toll Like Receptor 6	Protein Coding	43	GC04M038828	0.21
1028	TNXB	Tenascin XB	Protein Coding	43	GC06M032383	0.21
1029	UBC	Ubiquitin C	Protein Coding	43	GC12M124911	0.21
1030	TREH	Trehalase	Protein Coding	43	GC11M118657	0.21
1031	LIPG	Lipase G, Endothelial Type	Protein Coding	43	GC18P049560	0.21
1032	SELENBP1	Selenium Binding Protein 1	Protein Coding	43	GC01M151364	0.21
1033	OSM	Oncostatin M	Protein Coding	43	GC22M030262	0.21
1034	SDC1	Syndecan 1	Protein Coding	43	GC02M020200	0.21
1035	CDA	Cytidine Deaminase	Protein Coding	43	GC01P020588	0.21
1036	HSPA1A	Heat Shock Protein Family A (Hsp70) Member 1A	Protein Coding	43	GC06P033429	0.21
1037	IGFBP1	Insulin Like Growth Factor Binding Protein 1	Protein Coding	43	GC07P046473	0.21
1038	CD226	CD226 Molecule	Protein Coding	43	GC18M069831	0.21
1039	NDUFA13	NADH:Ubiquinone Oxidoreductase Subunit A13	Protein Coding	43	GC19P019515	0.21
1040	FCER1A	Fc Fragment Of IgE Receptor Ia	Protein Coding	43	GC01P159259	0.21
1041	ATG16L1	Autophagy Related 16 Like 1	Protein Coding	43	GC02P233215	0.21
1042	ADH1B	Alcohol Dehydrogenase 1B (Class I), Beta Polypeptide	Protein Coding	43	GC04M099304	0.21
1043	CNTF	Ciliary Neurotrophic Factor	Protein Coding	42	GC11P058622	0.21
1044	ITGA1	Integrin Subunit Alpha 1	Protein Coding	42	GC05P052788	0.21
1045	PRPF31	Pre-MRNA Processing Factor 31	Protein Coding	42	GC19P054165	0.21
1046	PTPRT	Protein Tyrosine Phosphatase Receptor Type T	Protein Coding	42	GC20M042072	0.21
1047	TNFRSF6B	TNF Receptor Superfamily Member 6b	Protein Coding	42	GC20P063696	0.21
1048	NR2C2	Nuclear Receptor Subfamily 2 Group C Member 2	Protein Coding	42	GC03P014947	0.21
1049	CLCF1	Cardiotrophin Like Cytokine Factor 1	Protein Coding	42	GC11M067364	0.21
1050	EPO	Erythropoietin	Protein Coding	42	GC07P100720	0.21
1051	GDF15	Growth Differentiation Factor 15	Protein Coding	42	GC19P022252	0.21
1052	EEF1B2	Eukaryotic Translation Elongation Factor 1 Beta 2	Protein Coding	42	GC02P206159	0.21
1053	DNTT	DNA Nucleotidylexotransferase	Protein Coding	42	GC10P096304	0.21
1054	MTRR	5-Methyltetrahydrofolate-Homocysteine Methyltransferase Reductase	Protein Coding	42	GC05P007851	0.21
1055	APBA2	Amyloid Beta Precursor Protein Binding Family A Member 2	Protein Coding	42	GC15P028888	0.21
1056	ADAM8	ADAM Metallopeptidase Domain 8	Protein Coding	42	GC10M133262	0.21
1057	ACTC1	Actin Alpha Cardiac Muscle 1	Protein Coding	42	GC15M034788	0.21
1058	BACH2	BTB Domain And CNC Homolog 2	Protein Coding	42	GC06M089926	0.21
1059	CTHRC1	Collagen Triple Helix Repeat Containing 1	Protein Coding	41	GC08P103371	0.21
1060	CLIC1	Chloride Intracellular Channel 1	Protein Coding	41	GC06M032375	0.21
1061	HIC1	HIC ZBTB Transcriptional Repressor 1	Protein Coding	41	GC17P002054	0.21
1062	COPA	COPI Coat Complex Subunit Alpha	Protein Coding	41	GC01M160288	0.21
1063	PRDM2	PR/SET Domain 2	Protein Coding	41	GC01P013700	0.21
1064	KIF2A	Kinesin Family Member 2A	Protein Coding	41	GC05P062306	0.21
1065	RECK	Reversion Inducing Cysteine Rich Protein With Kazal Motifs	Protein Coding	41	GC09P036036	0.21
1066	UGT1A6	UDP Glucuronosyltransferase Family 1 Member A6	Protein Coding	41	GC02P233691	0.21
1067	TEP1	Telomerase Associated Protein 1	Protein Coding	41	GC14M020365	0.21
1068	TERF2	Telomeric Repeat Binding Factor 2	Protein Coding	41	GC16M069355	0.21
1069	SCTR	Secretin Receptor	Protein Coding	41	GC02M119439	0.21
1070	F2RL2	Coagulation Factor II Thrombin Receptor Like 2	Protein Coding	41	GC05M076615	0.21
1071	CCR9	C-C Motif Chemokine Receptor 9	Protein Coding	41	GC03P045903	0.21
1072	CALB2	Calbindin 2	Protein Coding	41	GC16P071392	0.21
1073	MX1	MX Dynamin Like GTPase 1	Protein Coding	41	GC21P041420	0.21
1074	GPR55	G Protein-Coupled Receptor 55	Protein Coding	40	GC02M230907	0.21
1075	PIWIL1	Piwi Like RNA-Mediated Gene Silencing 1	Protein Coding	40	GC12P130337	0.21

1076	IL32	Interleukin 32	Protein Coding	40	GC16P004058	0.21
1077	ZNF148	Zinc Finger Protein 148	Protein Coding	40	GC03M125225	0.21
1078	FUCA2	Alpha-L-Fucosidase 2	Protein Coding	40	GC06M143494	0.21
1079	ATP4B	ATPase H+/K+ Transporting Subunit Beta	Protein Coding	40	GC13M113648	0.21
1080	CTF1	Cardiotrophin 1	Protein Coding	39	GC16P030910	0.21
1081	IL27	Interleukin 27	Protein Coding	39	GC16M028511	0.21
1082	KLF3	Kruppel Like Factor 3	Protein Coding	39	GC04P038668	0.21
1083	TRAK1	Trafficking Kinesin Protein 1	Protein Coding	39	GC03P042016	0.21
1084	ZNRD1	Zinc Ribbon Domain Containing 1	Protein Coding	39	GC06P033214	0.21
1085	ORMDL3	ORMDL Sphingolipid Biosynthesis Regulator 3	Protein Coding	39	GC17M039921	0.21
1086	SLC26A6	Solute Carrier Family 26 Member 6	Protein Coding	39	GC03M048625	0.21
1087	IFNA1	Interferon Alpha 1	Protein Coding	39	GC09P021478	0.21
1088	NAPA	NSF Attachment Protein Alpha	Protein Coding	39	GC19M047489	0.21
1089	NOVA1	NOVA Alternative Splicing Regulator 1	Protein Coding	39	GC14M026443	0.21
1090	ETV5	ETS Variant Transcription Factor 5	Protein Coding	39	GC03M186046	0.21
1091	ANKRD26	Ankyrin Repeat Domain 26	Protein Coding	39	GC10M026961	0.21
1092	GLG1	Golgi Glycoprotein 1	Protein Coding	38	GC16M074448	0.21
1093	UBASH3A	Ubiquitin Associated And SH3 Domain Containing A	Protein Coding	38	GC21P042403	0.21
1094	IL18RAP	Interleukin 18 Receptor Accessory Protein	Protein Coding	38	GC02P102418	0.21
1095	OLFM4	Olfactomedin 4	Protein Coding	38	GC13P053028	0.21
1096	BLZF1	Basic Leucine Zipper Nuclear Factor 1	Protein Coding	38	GC01P169367	0.21
1097	ASMT	Acetylserotonin O-Methyltransferase	Protein Coding	38	GC0XP001595	0.21
1098	AANAT	Aralkylamine N-Acetyltransferase	Protein Coding	38	GC17P076453	0.21
1099	IL1RAPL2	Interleukin 1 Receptor Accessory Protein Like 2	Protein Coding	37	GC0XP104566	0.21
1100	UCN3	Urocortin 3	Protein Coding	37	GC10P005396	0.21
1101	DNAH8	Dynein Axonemal Heavy Chain 8	Protein Coding	37	GC06P046078	0.21
1102	ATOH1	Atonal BHLH Transcription Factor 1	Protein Coding	37	GC04P093828	0.21
1103	LACTB	Lactamase Beta	Protein Coding	36	GC15P071910	0.21
1104	KLHL6	Kelch Like Family Member 6	Protein Coding	36	GC03M183487	0.21
1105	IKZF4	IKAROS Family Zinc Finger 4	Protein Coding	36	GC12P056007	0.21
1106	CLEC16A	C-Type Lectin Domain Containing 16A	Protein Coding	36	GC16P010944	0.21
1107	POMGNT2	Protein O-Linked Mannose N-Acetylglucosaminyltransferase 2 (Beta 1,4-)	Protein Coding	36	GC03M043121	0.21
1108	G3BP2	G3BP Stress Granule Assembly Factor 2	Protein Coding	36	GC04M075642	0.21
1109	MAGEA4	MAGE Family Member A4	Protein Coding	36	GC0XP151912	0.21
1110	MUC16	Mucin 16, Cell Surface Associated	Protein Coding	36	GC19M008848	0.21
1111	RILP	Rab Interacting Lysosomal Protein	Protein Coding	36	GC17M001646	0.21
1112	FCRL3	Fc Receptor Like 3	Protein Coding	36	GC01M157674	0.21
1113	IRX2	Iroquois Homeobox 2	Protein Coding	35	GC05M002745	0.21
1114	TADA3	Transcriptional Adaptor 3	Protein Coding	35	GC03M009779	0.21
1115	ZPBP2	Zona Pellucida Binding Protein 2	Protein Coding	35	GC17P039869	0.21
1116	FOXD1	Forkhead Box D1	Protein Coding	35	GC05M073444	0.21
1117	PSMG2	Proteasome Assembly Chaperone 2	Protein Coding	34	GC18P013073	0.21
1118	RABEP2	Rabaptin, RAB GTPase Binding Effector Protein 2	Protein Coding	34	GC16M028915	0.21
1119	ADAD1	Adenosine Deaminase Domain Containing 1	Protein Coding	34	GC04P122378	0.21
1120	FAM114A1	Family With Sequence Similarity 114 Member A1	Protein Coding	34	GC04P038867	0.21
1121	CSN1S1	Casein Alpha S1	Protein Coding	33	GC04P069932	0.21
1122	UCN2	Urocortin 2	Protein Coding	33	GC03M048561	0.21
1123	DDX53	DEAD-Box Helicase 53	Protein Coding	33	GC0XP022999	0.21
1124	IGKC	Immunoglobulin Kappa Constant	Protein Coding	32	GC02M089081	0.21
1125	SLFN12L	Schlafen Family Member 12 Like	Protein Coding	32	GC17M035464	0.21
1126	SELENOS	Selenoprotein S	Protein Coding	31	GC15M102816	0.21
1127	CTAG1B	Cancer/Testis Antigen 1B	Protein Coding	30	GC0XM154617	0.21
1128	RASSF10	Ras Association Domain Family Member 10	Protein Coding	30	GC11P012990	0.21
1129	INS-IGF2	INS-IGF2 Readthrough	Protein Coding	27	GC11M002177	0.21
1130	IGF2-AS	IGF2 Antisense RNA	RNA Gene	24	GC11P002140	0.21
1131	MIR1915	MicroRNA 1915	RNA Gene	15	GC10M021496	0.21
1132	MIR490	MicroRNA 490	RNA Gene	14	GC07P136903	0.21
1133	MIR3178	MicroRNA 3178	RNA Gene	13	GC16M002531	0.21
1134	MIR329-1	MicroRNA 329-1	RNA Gene	12	GC14P104444	0.21
1135	MIR329-2	MicroRNA 329-2	RNA Gene	12	GC14P104445	0.21
1136	LINC02864	Long Intergenic Non-Protein Coding RNA 2864	RNA Gene	9	GC18M073103	0.21
1137	TMEM132D-	TMEM132D Antisense RNA 1	RNA Gene	8	GC12P129110	0.21
1138	RPS14P1	Ribosomal Protein S14 Pseudogene 1	Pseudogene	5	GC01M206695	0.21
