# Supplementary material for: Pharmacological Effects of Agastache rugosa against Gastritis Using a Network Pharmacology Approach
Source: Biomolecules. 2020 Sep 9;10(9):1298. doi: 10.3390/biom10091298 (PMC7565599; doi:10.3390/biom10091298)
Supplement: Supplementary file 1 [file biomolecules-10-01298-s001.zip › gastritis_supplementary_table4.pdf]

Supplemenatary table 4. Protein-protein interactions in STITCH DB

node1	node2	node1_string_internal_id	node2_string_internal_id	combined_score
MAPK9	JUND	1859420	1844164	0.999
MTOR	RPS6KB1	1854147	1843035	0.999
MTOR	MAPKAP1	1854147	1845827	0.999
CDCA8	BIRC5	1849847	1848314	0.999
HSP90AA1	TP53	1851814	1846083	0.999
AURKB	INCENP	1863844	1858231	0.999
CTNNB1	EP300	1852818	1845316	0.999
CTNNB1	ERBB2	1852818	1846101	0.999
SHC1	CBL	1860266	1845465	0.999
HSP90AA1	EGFR	1851814	1846445	0.999
TP53	CDKN1A	1846083	1843736	0.999
EIF4EBP1	AKT1	1852358	1846136	0.999
AKT1S1	RPTOR	1852495	1848942	0.999
NOS3	AKT1	1847875	1846136	0.999
EGFR	STAT3	1846445	1845584	0.999
MTOR	AKT1S1	1854147	1852495	0.999
EP300	SMAD2	1845316	1845079	0.999
BCL2L11	BCL2L1	1858084	1848421	0.999
RICTOR	EIF4EBP1	1853522	1852358	0.999
JUN	MAPK3	1855436	1845265	0.999
BCAR1	PTK2	1859585	1852426	0.999
EGFR	CDH1	1846445	1844988	0.999
BRCA1	TP53	1861387	1846083	0.999
MDM2	EP300	1861282	1845316	0.999
MAPK9	JUN	1859420	1855436	0.999
MAPK8	TP53	1853988	1846083	0.999
PTK2	GRB2	1852426	1852164	0.999
HSP90AA1	NOS3	1851814	1847875	0.999
RICTOR	RPS6KB1	1853522	1843035	0.999
CASP9	CASP3	1851230	1849375	0.999
CTNNB1	EGFR	1852818	1846445	0.999
JUN	EP300	1855436	1845316	0.999
GRB2	ERBB2	1852164	1846101	0.999
EZH2	EED	1850211	1845338	0.999
MTOR	RHEB	1854147	1845084	0.999
TP53	EP300	1846083	1845316	0.999
MAPK8	FOS	1853988	1848822	0.999
HSP90AA1	PPP5C	1851814	1842173	0.999
AKT1	EP300	1846136	1845316	0.999
MLST8	RPTOR	1858459	1848942	0.999
JUN	MAPK8	1855436	1853988	0.999
MDM2	TP53	1861282	1846083	0.999
HSP90AA1	AHSA1	1851814	1842603	0.999
EIF4EBP1	MAPKAP1	1852358	1845827	0.999
ATM	TP53	1846577	1846083	0.999
SHC1	KDR	1860266	1845437	0.999
FOXO1	AKT1	1857055	1846136	0.999
JUN	FOS	1855436	1848822	0.999
JUN	ATF2	1855436	1845482	0.999
JUN	MAPK10	1855436	1853804	0.999
IKBKG	NFKBIA	1855067	1842621	0.999

MAPK8	JUND	1853988	1844164	0.999
FKBP1A	TGFBR1	1857423	1856216	0.999
APAF1	BCL2L1	1862487	1848421	0.999
EZH2	SUZ12	1850211	1849881	0.999
AKT1S1	MAPKAP1	1852495	1845827	0.999
CTNND1	EGFR	1858683	1846445	0.999
SHC1	ERBB2	1860266	1846101	0.999
MTOR	AKT1	1854147	1846136	0.999
SUZ12	EED	1849881	1845338	0.999
RICTOR	AKT1	1853522	1846136	0.999
MAPK9	ATF2	1859420	1845482	0.999
AKT1	MAPKAP1	1846136	1845827	0.999
MDM2	CDKN1A	1861282	1843736	0.999
MLST8	MTOR	1858459	1854147	0.999
CTNNB1	CDH1	1852818	1844988	0.999
INCENP	BIRC5	1858231	1848314	0.999
APAF1	CASP9	1862487	1851230	0.999
IKBKB	NFKBIA	1861846	1842621	0.999
HSP90AA1	CDC37	1851814	1842879	0.999
INCENP	CDCA8	1858231	1849847	0.999
SHC1	EGFR	1860266	1846445	0.999
MLST8	AKT1S1	1858459	1852495	0.999
IGFBP3	IGF1	1857331	1848439	0.999
STAT1	EGFR	1854113	1846445	0.999
GRB2	EGFR	1852164	1846445	0.999
AKT1	CDKN1A	1846136	1843736	0.999
EGFR	ERBB2	1846445	1846101	0.999
MLST8	EIF4EBP1	1858459	1852358	0.999
RICTOR	AKT1S1	1853522	1852495	0.999
NFE2L2	KEAP1	1858451	1842313	0.999
STAT1	EP300	1854113	1845316	0.999
EGFR	CBL	1846445	1845465	0.999
HSP90AA1	ERBB2	1851814	1846101	0.999
KAT2B	EP300	1845408	1845316	0.999
HSP90AA1	STIP1	1851814	1848791	0.999
HSP90AA1	AKT1	1851814	1846136	0.999
PPARG	EP300	1847090	1845316	0.999
MTOR	STAT3	1854147	1845584	0.999
FOXH1	SMAD2	1856663	1845079	0.999
JUN	ATF3	1855436	1852803	0.999
AURKB	CDCA8	1863844	1849847	0.999
HSP90AA1	PTGES3	1851814	1845046	0.999
MTOR	RICTOR	1854147	1853522	0.999
RICTOR	MAPKAP1	1853522	1845827	0.999
STAT1	STAT3	1854113	1845584	0.999
VEGFA	FLT1	1855611	1846791	0.999
IGF2	IGFBP3	1859600	1857331	0.999
GRB2	CBL	1852164	1845465	0.999
FKBP1A	MTOR	1857423	1854147	0.999
MAPK9	TP53	1859420	1846083	0.999
FOS	JUND	1848822	1844164	0.999
MLST8	RICTOR	1858459	1853522	0.999
IKBKB	IKBKG	1861846	1855067	0.999
BCL2L1	TP53	1848421	1846083	0.999

MDM2	AKT1	1861282	1846136	0.999
MTOR	EIF4EBP1	1854147	1852358	0.999
BRCA1	ATM	1861387	1846577	0.999
TGFBR1	SMAD2	1856216	1845079	0.999
AURKB	BIRC5	1863844	1848314	0.999
TP53	KAT2B	1846083	1845408	0.999
BCAR1	PXN	1859585	1843137	0.999
RPTOR	RHEB	1848942	1845084	0.999
EIF4EBP1	RPTOR	1852358	1848942	0.999
SHC1	GRB2	1860266	1852164	0.999
MTOR	RPTOR	1854147	1848942	0.999
STAT3	EP300	1845584	1845316	0.999
MAPK8	ATF2	1853988	1845482	0.999
PTK2	PXN	1852426	1843137	0.999
RPTOR	RPS6KB1	1848942	1843035	0.999
CTNND1	CDH1	1858683	1844988	0.999
CTNNB1	AKT1	1852818	1846136	0.999
VEGFA	KDR	1855611	1845437	0.999
SUGT1	HSP90AA1	1856790	1851814	0.999
TP53	MAPK1	1846083	1842512	0.998
MDM2	ATM	1861282	1846577	0.998
CASP9	BIRC5	1851230	1848314	0.998
MLST8	MAPKAP1	1858459	1845827	0.998
TLR4	HMGB1	1856025	1852653	0.998
ERBB2	STAT3	1846101	1845584	0.998
PTK2	TP53	1852426	1846083	0.998
BCL2L1	AKT1	1848421	1846136	0.998
MAPK10	JUND	1853804	1844164	0.998
TP53	SMAD2	1846083	1845079	0.998
MAPK8	CDKN1A	1853988	1843736	0.998
RICTOR	RPTOR	1853522	1848942	0.998
JUN	MAPK1	1855436	1842512	0.998
ATF3	TP53	1852803	1846083	0.998
JUN	CDKN1A	1855436	1843736	0.997
IKBKG	ATM	1855067	1846577	0.997
APAF1	CASP3	1862487	1849375	0.997
FOS	MAPK1	1848822	1842512	0.997
MAPK9	FOS	1859420	1848822	0.997
AKT1	RPS6KB1	1846136	1843035	0.997
EIF4EBP1	RPS6KB1	1852358	1843035	0.997
RPTOR	AKT1	1848942	1846136	0.997
GRB2	KDR	1852164	1845437	0.997
MAPK10	ATF2	1853804	1845482	0.997
ATF3	ATF2	1852803	1845482	0.997
BCL2L11	MAPK8	1858084	1853988	0.997
JUN	TP53	1855436	1846083	0.997
EGFR	TP53	1846445	1846083	0.997
RHEB	RPS6KB1	1845084	1843035	0.997
APAF1	TP53	1862487	1846083	0.996
IKBKB	AKT1	1861846	1846136	0.996
MAPK8	BCL2L1	1853988	1848421	0.996
STIP1	AHSA1	1848791	1842603	0.996
STAT1	FOS	1854113	1848822	0.996
MAPK8	PXN	1853988	1843137	0.996

CTNND1	CTNNB1	1858683	1852818	0.996
SHC1	FLT1	1860266	1846791	0.996
FOS	EP300	1848822	1845316	0.996
MMP9	JUN	1855661	1855436	0.996
EP300	JUND	1845316	1844164	0.996
PXN	MAPK1	1843137	1842512	0.996
AKT1S1	AKT1	1852495	1846136	0.996
EP300	CDKN1A	1845316	1843736	0.996
FOS	TP53	1848822	1846083	0.996
KAT2B	SMAD2	1845408	1845079	0.996
MAPK10	FOS	1853804	1848822	0.995
NFE2L2	JUN	1858451	1855436	0.995
SHC1	IL2	1860266	1843096	0.995
HSP90AA1	KDR	1851814	1845437	0.995
MAPK3	MAPK1	1845265	1842512	0.994
HMGB1	TP53	1852653	1846083	0.994
STAT3	CDKN1A	1845584	1843736	0.994
BCAR1	GRB2	1859585	1852164	0.994
MDM2	KAT2B	1861282	1845408	0.994
MDM2	CASP3	1861282	1849375	0.994
FOXO1	MAPK8	1857055	1853988	0.994
JUN	NOS3	1855436	1847875	0.993
IGF1	RPS6KB1	1848439	1843035	0.993
MTOR	STAT1	1854147	1854113	0.993
CBL	KDR	1845465	1845437	0.993
ATF2	MAPK1	1845482	1842512	0.993
FOS	IL2	1848822	1843096	0.993
JUN	IL2	1855436	1843096	0.993
VEGFA	IGF1	1855611	1848439	0.993
FOXO1	STAT3	1857055	1845584	0.993
FOXO1	EP300	1857055	1845316	0.993
MAPK8	STAT3	1853988	1845584	0.993
MLST8	RPS6KB1	1858459	1843035	0.993
CASP9	AKT1	1851230	1846136	0.993
PTK2	ERBB2	1852426	1846101	0.993
FOXO1	CTNNB1	1857055	1852818	0.993
MAPK9	BCL2L11	1859420	1858084	0.993
IGFBP3	TP53	1857331	1846083	0.993
SHC1	IGF1	1860266	1848439	0.992
CASP3	CDKN1A	1849375	1843736	0.992
PTK2	STAT3	1852426	1845584	0.992
MTOR	HSP90AA1	1854147	1851814	0.992
CASP9	BCL2L1	1851230	1848421	0.992
JUN	CTNNB1	1855436	1852818	0.992
BRCA1	EP300	1861387	1845316	0.992
SHC1	MAPK1	1860266	1842512	0.991
MMP9	VEGFA	1855661	1855611	0.991
APAF1	HSP90AA1	1862487	1851814	0.991
ATF2	MAPK3	1845482	1845265	0.991
JUN	STAT3	1855436	1845584	0.991
FOS	ATF2	1848822	1845482	0.991
IL5	IL2	1843289	1843096	0.991
EIF4EBP1	RHEB	1852358	1845084	0.991
CASP3	CDH1	1849375	1844988	0.991

VEGFA	EGFR	1855611	1846445	0.991
TP53	MAPK3	1846083	1845265	0.99
HSP90AA1	MAPK1	1851814	1842512	0.99
STAT1	IL2	1854113	1843096	0.99
FOS	MAPK3	1848822	1845265	0.99
AGT	FOS	1854424	1848822	0.99
FOXO1	CDKN1A	1857055	1843736	0.99
MAPK3	PXN	1845265	1843137	0.989
STAT3	MAPK1	1845584	1842512	0.989
BCL2L11	JUN	1858084	1855436	0.989
JUN	KAT2B	1855436	1845408	0.989
IGF2	VEGFA	1859600	1855611	0.989
JUN	IL5	1855436	1843289	0.988
MAPK8	IL2	1853988	1843096	0.988
MTOR	TP53	1854147	1846083	0.988
IGF1	CDH1	1848439	1844988	0.988
VEGFA	ERBB2	1855611	1846101	0.988
HMGB1	KAT2B	1852653	1845408	0.988
JUN	STAT1	1855436	1854113	0.987
SHC1	PTK2	1860266	1852426	0.987
GRB2	AKT1	1852164	1846136	0.987
SHC1	AKT1	1860266	1846136	0.987
EIF4EBP1	MAPK1	1852358	1842512	0.987
IGF1	EGFR	1848439	1846445	0.987
PTK2	FLT1	1852426	1846791	0.986
NOS3	KDR	1847875	1845437	0.986
MTOR	EGFR	1854147	1846445	0.986
GRB2	MAPK1	1852164	1842512	0.986
STIP1	PTGES3	1848791	1845046	0.985
MAPK3	SMAD2	1845265	1845079	0.985
MLST8	RHEB	1858459	1845084	0.985
MMP9	LCN2	1855661	1846529	0.985
IGFBP3	MAPK8	1857331	1853988	0.984
EIF4EBP1	ATM	1852358	1846577	0.984
SMAD2	MAPK1	1845079	1842512	0.984
PPARG	IL2	1847090	1843096	0.984
SHC1	VEGFA	1860266	1855611	0.984
FLT1	KDR	1846791	1845437	0.983
SHC1	MAPK8	1860266	1853988	0.983
EGFR	CDC37	1846445	1842879	0.983
AURKB	TP53	1863844	1846083	0.982
VEGFA	AKT1	1855611	1846136	0.982
FOS	IL1B	1848822	1845335	0.981
JUN	IL1B	1855436	1845335	0.981
TGFBR1	CTNNB1	1856216	1852818	0.98
BCAR1	CBL	1859585	1845465	0.98
STAT1	ERBB2	1854113	1846101	0.98
CTNNB1	KDR	1852818	1845437	0.98
SHC1	AGT	1860266	1854424	0.98
JUN	BCL2L1	1855436	1848421	0.979
FOXO1	RPS6KB1	1857055	1843035	0.979
FKBP1A	RPTOR	1857423	1848942	0.978
IKBKB	HSP90AA1	1861846	1851814	0.978
CASP3	BIRC5	1849375	1848314	0.978

SUGT1	PTGES3	1856790	1845046	0.978
EGFR	MAPK1	1846445	1842512	0.978
MLST8	AKT1	1858459	1846136	0.978
SMAD2	CDKN1A	1845079	1843736	0.978
ATF2	EP300	1845482	1845316	0.978
CASP3	AKT1	1849375	1846136	0.978
MAPK9	CTNNB1	1859420	1852818	0.978
STAT3	MAPK3	1845584	1845265	0.978
AKT1	CDC37	1846136	1842879	0.977
JUN	AKT1	1855436	1846136	0.977
FOXO1	SMAD2	1857055	1845079	0.977
VEGFA	HSP90AA1	1855611	1851814	0.977
STAT1	BCL2L1	1854113	1848421	0.976
BCL2L1	STAT3	1848421	1845584	0.976
MMP9	CDH1	1855661	1844988	0.976
GRB2	FLT1	1852164	1846791	0.976
GRB2	MAPK3	1852164	1845265	0.976
IKBKB	KEAP1	1861846	1842313	0.976
PTK2	EGFR	1852426	1846445	0.976
EGFR	PXN	1846445	1843137	0.976
AKT1	TP53	1846136	1846083	0.975
VEGFA	EP300	1855611	1845316	0.975
BCAR1	MAPK8	1859585	1853988	0.975
STAT1	PTK2	1854113	1852426	0.975
CTNNB1	BIRC5	1852818	1848314	0.975
FOS	STAT3	1848822	1845584	0.975
AKT1	STAT3	1846136	1845584	0.974
ERBB2	TP53	1846101	1846083	0.974
ATM	CDKN1A	1846577	1843736	0.974
MTOR	BCL2L1	1854147	1848421	0.973
STAT1	AKT1	1854113	1846136	0.973
RPS6KB1	MAPK1	1843035	1842512	0.973
AKT1S1	RHEB	1852495	1845084	0.971
SHC1	MAPK3	1860266	1845265	0.971
MAPK8	ATF3	1853988	1852803	0.97
FLT1	CBL	1846791	1845465	0.97
HSP90AA1	RPS6KB1	1851814	1843035	0.97
GRB2	STAT3	1852164	1845584	0.969
FOXO1	MTOR	1857055	1854147	0.969
ATF3	JUND	1852803	1844164	0.969
ATM	ATF2	1846577	1845482	0.968
AGT	JUND	1854424	1844164	0.968
CTNNB1	SMAD2	1852818	1845079	0.967
JUN	SMAD2	1855436	1845079	0.967
MMP9	MAPK8	1855661	1853988	0.967
VEGFA	JUN	1855611	1855436	0.966
MAPK8	CTNNB1	1853988	1852818	0.965
FOS	SMAD2	1848822	1845079	0.965
TP53	STAT3	1846083	1845584	0.965
APAF1	AKT1	1862487	1846136	0.965
ERBB2	CBL	1846101	1845465	0.964
HMGB1	MAPK1	1852653	1842512	0.964
BIRC5	TP53	1848314	1846083	0.964
JUN	EGFR	1855436	1846445	0.964

BRCA1	AKT1	1861387	1846136	0.963
IGF1	AKT1	1848439	1846136	0.963
STAT3	IL2	1845584	1843096	0.962
BCL2L11	MAPK10	1858084	1853804	0.962
FN1	PXN	1853098	1843137	0.962
VEGFA	TP53	1855611	1846083	0.961
TP53	CDH1	1846083	1844988	0.961
CTNND1	ERBB2	1858683	1846101	0.961
MAPK8	MAPK1	1853988	1842512	0.961
IL13	IL5	1848685	1843289	0.961
GRB2	RPS6KB1	1852164	1843035	0.96
VEGFA	NOS3	1855611	1847875	0.96
BCL2L11	AKT1	1858084	1846136	0.96
HSP90AA1	STAT3	1851814	1845584	0.96
IGFBP3	EGFR	1857331	1846445	0.959
FOS	AKT1	1848822	1846136	0.959
HSP90AA1	BIRC5	1851814	1848314	0.959
JUN	NFKBIA	1855436	1842621	0.959
MTOR	GRB2	1854147	1852164	0.958
MMP9	CTNNB1	1855661	1852818	0.957
JUN	HMGB1	1855436	1852653	0.956
AKT1	IL2	1846136	1843096	0.956
IKBKB	TLR4	1861846	1856025	0.956
SHC1	MAPK9	1860266	1859420	0.956
BCL2L1	IL2	1848421	1843096	0.956
GRB2	IGF1	1852164	1848439	0.956
BIRC5	AKT1	1848314	1846136	0.956
JUND	MAPK1	1844164	1842512	0.955
FOXO1	MAPK10	1857055	1853804	0.955
PTK2	CBL	1852426	1845465	0.955
AKT1	CDH1	1846136	1844988	0.955
VEGFA	PTK2	1855611	1852426	0.954
EZH2	AKT1	1850211	1846136	0.954
MTOR	IL2	1854147	1843096	0.954
STAT1	TP53	1854113	1846083	0.954
MAPK8	PPARG	1853988	1847090	0.954
AGT	MAPK1	1854424	1842512	0.954
CTNNB1	FOS	1852818	1848822	0.953
ATF2	JUND	1845482	1844164	0.953
SUGT1	AHSA1	1856790	1842603	0.953
MAPK8	EP300	1853988	1845316	0.953
MMP9	AKT1	1855661	1846136	0.952
MAPK9	MAPK8	1859420	1853988	0.951
GRB2	IL2	1852164	1843096	0.951
EP300	CDH1	1845316	1844988	0.95
SHC1	HSP90AA1	1860266	1851814	0.95
TLR4	EGFR	1856025	1846445	0.95
MAPK10	TP53	1853804	1846083	0.95
BCL2L11	FOXO1	1858084	1857055	0.949
NFE2L2	FOS	1858451	1848822	0.949
MAPK8	ERBB2	1853988	1846101	0.949
MDM2	HSP90AA1	1861282	1851814	0.949
STAT1	KDR	1854113	1845437	0.948
AGT	TP53	1854424	1846083	0.948

KDR	PXN	1845437	1843137	0.948
AKT1	KAT2B	1846136	1845408	0.948
TGFBR1	HSP90AA1	1856216	1851814	0.948
AKT1S1	EIF4EBP1	1852495	1852358	0.948
CTNNB1	IGF1	1852818	1848439	0.947
MMP9	EGFR	1855661	1846445	0.947
ERBB2	CDC37	1846101	1842879	0.947
EIF4EBP1	MAPK3	1852358	1845265	0.946
AGT	GRB2	1854424	1852164	0.946
TLR4	AKT1	1856025	1846136	0.945
MAPK9	MAPK10	1859420	1853804	0.945
VEGFA	STAT3	1855611	1845584	0.945
EZH2	STAT3	1850211	1845584	0.945
NFE2L2	PPARG	1858451	1847090	0.945
FOS	IL5	1848822	1843289	0.944
STAT1	MAPK3	1854113	1845265	0.944
BCL2L1	EGFR	1848421	1846445	0.944
IGF1	MAPK3	1848439	1845265	0.944
TLR4	IL1B	1856025	1845335	0.943
TLR4	STAT1	1856025	1854113	0.943
MMP9	TP53	1855661	1846083	0.942
TLR4	IKBKG	1856025	1855067	0.942
VEGFA	PXN	1855611	1843137	0.942
ERBB2	CDH1	1846101	1844988	0.942
AKT1S1	RPS6KB1	1852495	1843035	0.942
FOS	EGFR	1848822	1846445	0.942
BIRC5	STAT3	1848314	1845584	0.942
CBL	IL2	1845465	1843096	0.942
BIRC5	EGFR	1848314	1846445	0.941
BIRC5	ERBB2	1848314	1846101	0.941
HSP90AA1	KEAP1	1851814	1842313	0.94
PXN	RPS6KB1	1843137	1843035	0.94
AKT1	RHEB	1846136	1845084	0.94
MMP9	NOS3	1855661	1847875	0.94
FKBP1A	SMAD2	1857423	1845079	0.94
NOS3	EP300	1847875	1845316	0.94
NFKBIA	MAPK1	1842621	1842512	0.939
FOXO1	RICTOR	1857055	1853522	0.939
FOXH1	EP300	1856663	1845316	0.939
MMP9	IL1B	1855661	1845335	0.939
IGF2	CTNNB1	1859600	1852818	0.939
STAT1	MAPK1	1854113	1842512	0.938
VEGFA	GRB2	1855611	1852164	0.938
IKBKB	CDC37	1861846	1842879	0.938
FLT1	PXN	1846791	1843137	0.938
JUN	IL13	1855436	1848685	0.938
SMAD2	CDH1	1845079	1844988	0.937
TLR4	MAPK8	1856025	1853988	0.937
IKBKB	CTNNB1	1861846	1852818	0.937
EP300	NFKBIA	1845316	1842621	0.937
AGT	NOS3	1854424	1847875	0.937
NOS2	NFKBIA	1850906	1842621	0.936
JUN	SELE	1855436	1851385	0.936
IGF1	PXN	1848439	1843137	0.935

ATF2	KAT2B	1845482	1845408	0.934
IGF2	CDH1	1859600	1844988	0.933
HSP90AA1	IL2	1851814	1843096	0.933
MAPK9	IL2	1859420	1843096	0.933
FOS	IL13	1848822	1848685	0.933
MAPK3	NFKBIA	1845265	1842621	0.933
JUN	NOS2	1855436	1850906	0.933
VEGFA	FN1	1855611	1853098	0.932
STIP1	CDC37	1848791	1842879	0.931
TLR4	STAT3	1856025	1845584	0.931
IL2	RPS6KB1	1843096	1843035	0.931
BCAR1	KDR	1859585	1845437	0.93
IGF1	MAPK1	1848439	1842512	0.929
HMGB1	MAPK3	1852653	1845265	0.929
MMP9	STAT3	1855661	1845584	0.929
IKBKG	CDC37	1855067	1842879	0.929
GRB2	IL5	1852164	1843289	0.929
IL1B	IL2	1845335	1843096	0.928
FOS	BCL2L1	1848822	1848421	0.928
BCAR1	VEGFA	1859585	1855611	0.927
BCAR1	RPS6KB1	1859585	1843035	0.927
MMP9	FLT1	1855661	1846791	0.926
BIRC5	CDKN1A	1848314	1843736	0.926
FOXO1	IGF1	1857055	1848439	0.926
BCAR1	IGF1	1859585	1848439	0.926
IKBKB	HMGB1	1861846	1852653	0.925
VEGFA	CBL	1855611	1845465	0.925
PTK2	KDR	1852426	1845437	0.925
HMGB1	NFKBIA	1852653	1842621	0.924
IGFBP3	EP300	1857331	1845316	0.924
MAPK3	IL2	1845265	1843096	0.923
PPARG	TP53	1847090	1846083	0.923
IL2	MAPK1	1843096	1842512	0.923
JUN	IGF1	1855436	1848439	0.923
SHC1	TGFBR1	1860266	1856216	0.923
BCL2L11	CASP3	1858084	1849375	0.923
FOS	IGF1	1848822	1848439	0.922
MMP9	ERBB2	1855661	1846101	0.922
ATF2	IL2	1845482	1843096	0.922
IL1B	EP300	1845335	1845316	0.922
FN1	PTK2	1853098	1852426	0.922
BCL2L1	ERBB2	1848421	1846101	0.921
STAT3	CDH1	1845584	1844988	0.921
EZH2	TP53	1850211	1846083	0.921
FN1	IGF1	1853098	1848439	0.921
SUGT1	CDC37	1856790	1842879	0.921
MAPK3	CDH1	1845265	1844988	0.921
CASP3	BCL2L1	1849375	1848421	0.921
IL13	IL2	1848685	1843096	0.921
BCL2L11	FOS	1858084	1848822	0.92
CDH1	MAPK1	1844988	1842512	0.92
IGF2	FN1	1859600	1853098	0.92
IGF2	IGF1	1859600	1848439	0.919
MAPK9	GRB2	1859420	1852164	0.919

AKT1	SMAD2	1846136	1845079	0.918
NOS2	ATF2	1850906	1845482	0.918
BCAR1	FLT1	1859585	1846791	0.917
MMP9	JUND	1855661	1844164	0.917
AKT1	ATF2	1846136	1845482	0.916
CBL	PXN	1845465	1843137	0.916
HMGB1	ATF2	1852653	1845482	0.915
BRCA1	ATF2	1861387	1845482	0.915
CASP3	TP53	1849375	1846083	0.914
ERBB2	CDKN1A	1846101	1843736	0.914
TLR4	MMP9	1856025	1855661	0.913
JUN	CDH1	1855436	1844988	0.913
NOS3	TP53	1847875	1846083	0.913
VEGFA	CTNNB1	1855611	1852818	0.912
IGF1	STAT3	1848439	1845584	0.912
IGF1	NOS3	1848439	1847875	0.912
IGFBP3	AKT1	1857331	1846136	0.912
MTOR	CTNNB1	1854147	1852818	0.912
CTNNB1	STAT3	1852818	1845584	0.912
IGF1	ERBB2	1848439	1846101	0.912
NOS3	STAT3	1847875	1845584	0.91
MTOR	PPARG	1854147	1847090	0.909
AKT1	PTGES3	1846136	1845046	0.909
IGF1	KDR	1848439	1845437	0.909
STAT1	CDKN1A	1854113	1843736	0.909
RPTOR	MAPK1	1848942	1842512	0.909
VEGFA	PPARG	1855611	1847090	0.909
MAPK3	JUND	1845265	1844164	0.908
EZH2	CDH1	1850211	1844988	0.908
BCAR1	FN1	1859585	1853098	0.907
TGFBR1	RPS6KB1	1856216	1843035	0.907
CTNNB1	CASP3	1852818	1849375	0.906
JUN	JUND	1855436	1844164	0.906
INCENP	CTNNB1	1858231	1852818	0.905
CTNNB1	FLT1	1852818	1846791	0.905
NOS2	NOS3	1850906	1847875	0.905
SELE	ATF2	1851385	1845482	0.903
STAT3	IL1B	1845584	1845335	0.903
CTNND1	IGF1	1858683	1848439	0.903
PPARG	IL1B	1847090	1845335	0.903
VEGFA	IL1B	1855611	1845335	0.903
MTOR	PTGES3	1854147	1845046	0.902
FOS	PPARG	1848822	1847090	0.901
SHC1	CDC37	1860266	1842879	0.901
IKBKB	MTOR	1861846	1854147	0.901
MAPK8	RPS6KB1	1853988	1843035	0.901
FOXO1	EGFR	1857055	1846445	0.901
MAPK8	MAPK10	1853988	1853804	0.901
PTGES3	IL2	1845046	1843096	0.9
IGF2	CTNND1	1859600	1858683	0.9
SHC1	TP53	1860266	1846083	0.9
PTGES3	RPS6KB1	1845046	1843035	0.9
CCR3	AGT	1862185	1854424	0.9
